# Supplementary material for: A single-cell atlas of Drosophila trachea reveals glycosylation-mediated Notch signaling in cell fate specification
Source: Nat Commun. 2024 Mar 6;15:2019. doi: 10.1038/s41467-024-46455-w (PMC10917797; doi:10.1038/s41467-024-46455-w)
Supplement: Supplementary file 1 — Supplementary Information [file 41467_2024_46455_MOESM1_ESM.pdf]

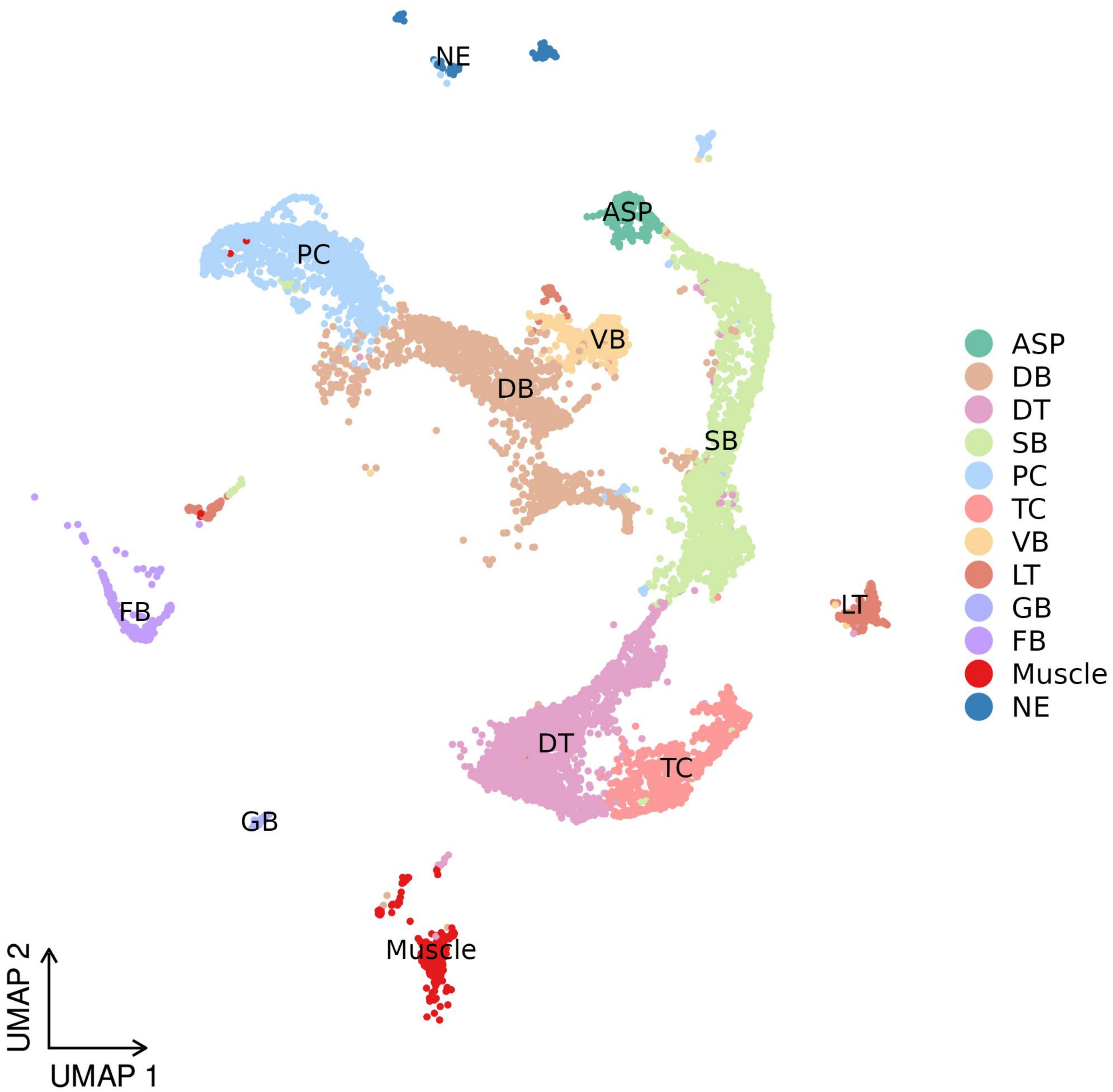

NE
ASP
PC
VB
DB
SB
FB
LT
DT
TC
GB
Muscle
ASP
DB
DT
SB
PC
TC
VB
LT
GB
FB
Muscle
NE
UMAP 2
UMAP 1

Supplementary Figure 1. A UMAP depicting annotated cell types. DT: dorsal trunk; DB: dorsal branch; TC: transverse connective; ASP: air sac primordium; VB: visceral branch; SB: spiracular branch; PC: progenitor cells; LT: lateral trunk; GB: ganglionic branches; NE: neuroendocrine cell; FB: fat body.

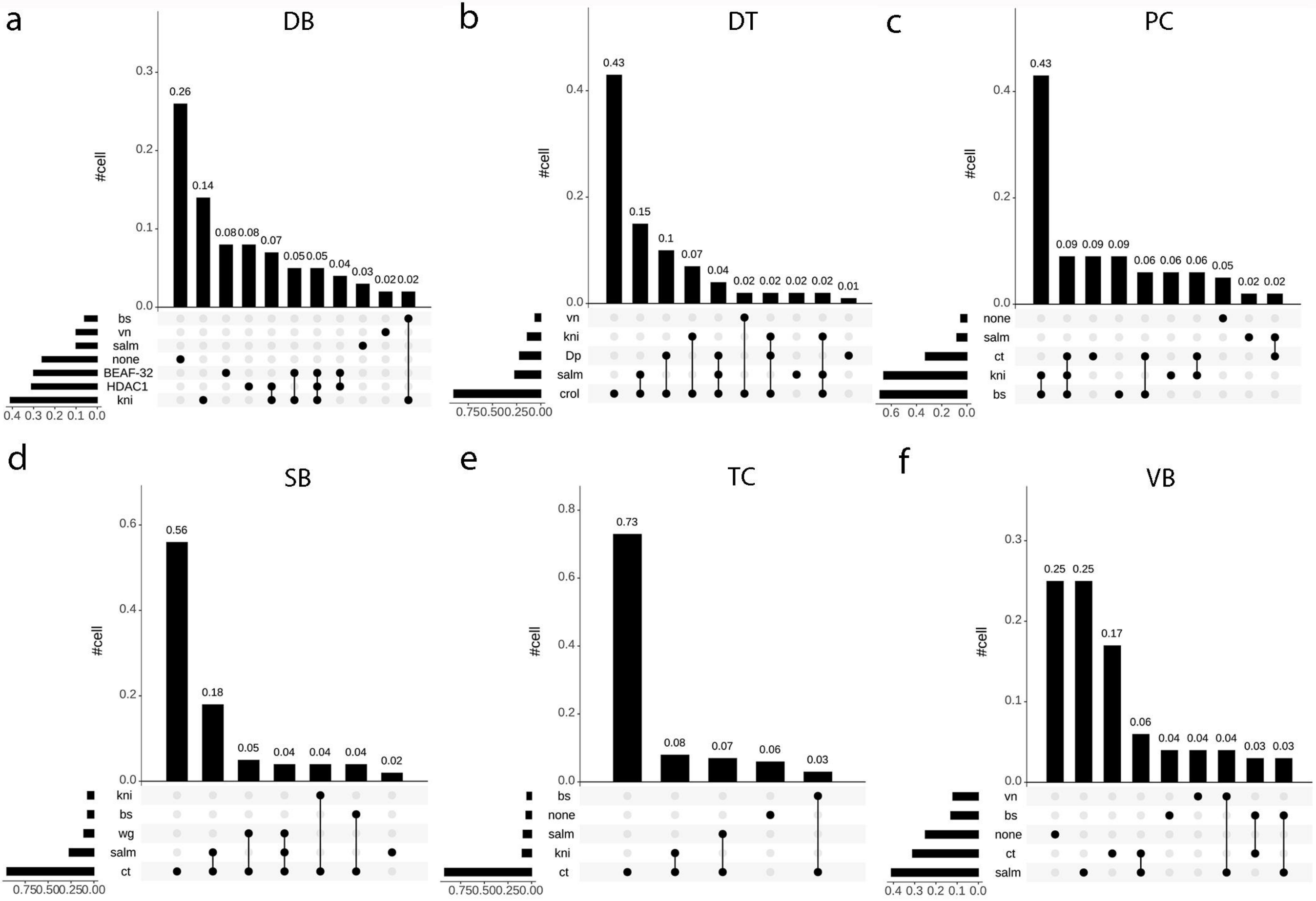
a
DB
#cell
0.26
0.14
0.08 0.08
0.07
0.05 0.05
0.04
0.03
0.02 0.02
bs
vn
salm
none
BEAF-32
HDAC1
kni
b
DT
0.43
0.15
0.1
0.07
0.04
0.02 0.02 0.02 0.02
0.01
vn
kni
Dp
salm
crol
c
PC
0.43
0.09 0.09 0.09
0.06 0.06 0.06
0.05
0.02 0.02
none
salm
ct
kni
bs
d
SB
0.56
0.18
0.05
0.04 0.04 0.04
0.02
kni
bs
wg
salm
ct
e
TC
0.73
0.08
0.07
0.06
0.03
bs
none
salm
kni
ct
f
VB
0.25 0.25
0.17
0.06
0.04 0.04 0.04
0.03 0.03
vn
bs
none
ct
salm

Supplementary Figure 2. Upset plots illustrating the expression of marker genes in each clusters. a, DB, **b**, DT, **c**, PC, **d**, SB, **e**, TC, **f**, VB.

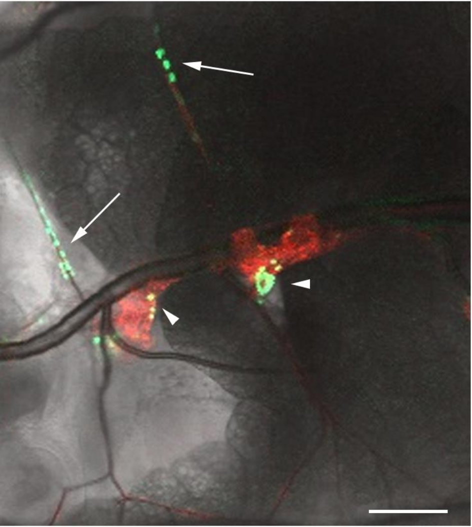

Supplementary Figure 3. Fucci fly illustrating the tracheal cells that are in mitotic cycle and are proliferation-competent. Arrows indicate DB. Arrowheads indicate PCs. Scale bar: 100 μm. Genotype: *btl-Gal4*/+; *P[B123]-RFP-moe*/*UAS-SG2M-Green*.

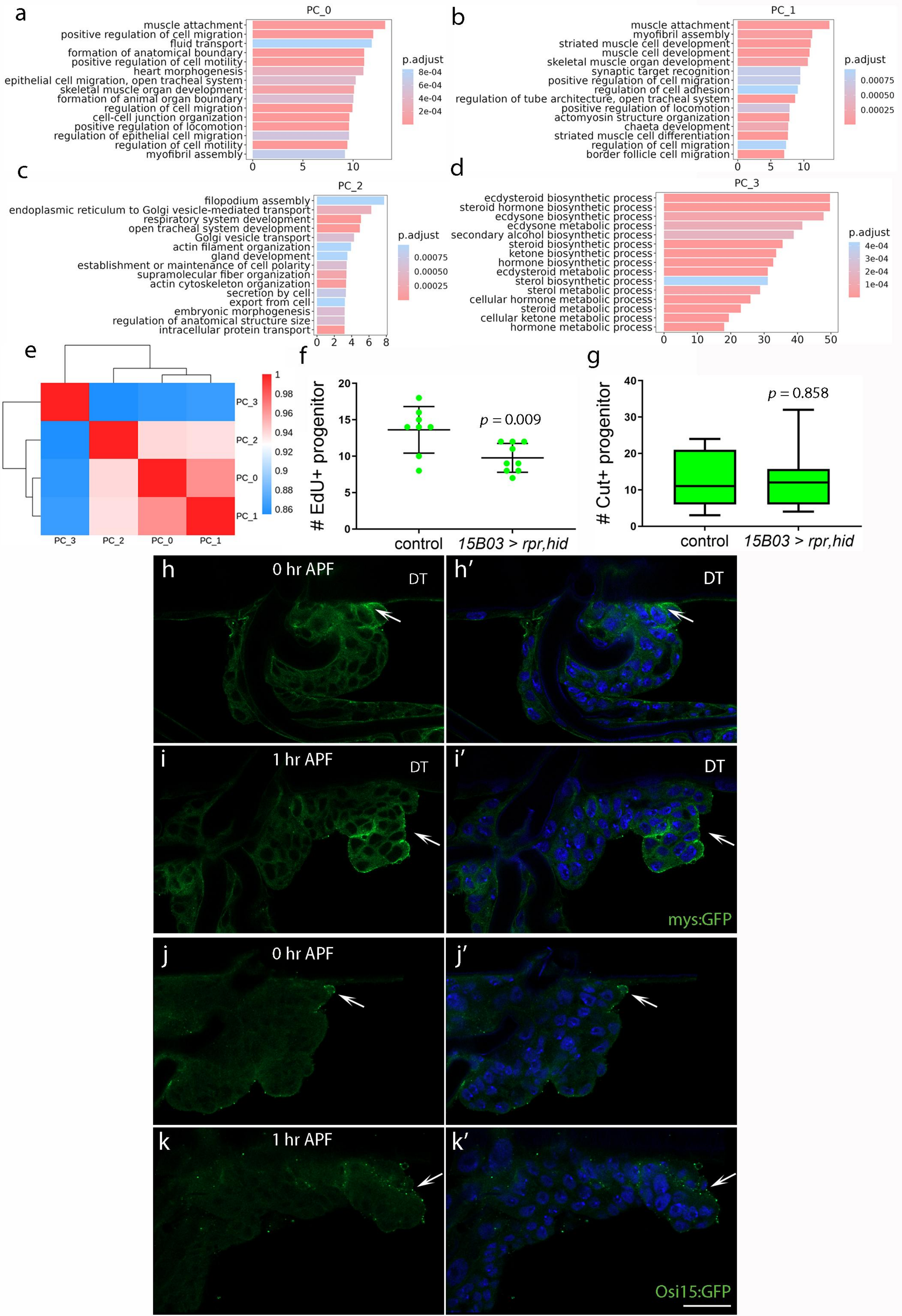

a
PC_0
muscle attachment
positive regulation of cell migration
fluid transport
formation of anatomical boundary
positive regulation of cell motility
heart morphogenesis
epithelial cell migration, open tracheal system
skeletal muscle organ development
formation of animal organ boundary
regulation of cell migration
cell-cell junction organization
positive regulation of locomotion
regulation of epithelial cell migration
regulation of cell motility
myofibril assembly
p.adjust
b
PC_1
muscle attachment
myofibril assembly
striated muscle cell development
muscle cell development
skeletal muscle organ development
synaptic target recognition
positive regulation of cell migration
regulation of cell adhesion
regulation of tube architecture, open tracheal system
positive regulation of locomotion
actomyosin structure organization
chaeta development
striated muscle cell differentiation
regulation of cell migration
border follicle cell migration
c
PC_2
filopodium assembly
endoplasmic reticulum to Golgi vesicle-mediated transport
respiratory system development
open tracheal system development
Golgi vesicle transport
actin filament organization
gland development
establishment or maintenance of cell polarity
supramolecular fiber organization
actin cytoskeleton organization
secretion by cell
export from cell
embryonic morphogenesis
regulation of anatomical structure size
intracellular protein transport
d
PC_3
ecdysteroid biosynthetic process
steroid hormone biosynthetic process
ecdysone biosynthetic process
ecdysone metabolic process
secondary alcohol biosynthetic process
steroid biosynthetic process
ketone biosynthetic process
hormone biosynthetic process
ecdysteroid metabolic process
sterol biosynthetic process
sterol metabolic process
cellular hormone metabolic process
steroid metabolic process
cellular ketone metabolic process
hormone metabolic process
e
f
# EdU+ progenitor
p = 0.009
control
15B03 > rpr,hid
g
# Cut+ progenitor
p = 0.858
control
15B03 > rpr,hid
h
0 hr APF
DT
h′
DT
i
1 hr APF
DT
i′
DT
mys:GFP
j
0 hr APF
j′
k
1 hr APF
k′
Osi15:GFP

Supplementary Figure 4. Subpopulations of tracheal progenitors. a-d, Bar graph depicting functional clusters among the marker genes in subclusters of progenitors. **e**, Correlation heatmap representing strength of relationships between subclusters of PC. **f**, Scatter plot showing the number of incorporated EdU foci in tracheal progenitors of control (*tub-Gal80^{ts}/+; 15B03-Gal4/+*) (*n* = 8) and muscle-perturbed flies (*UAS-rpr,hid/+; tub-Gal80^{ts}/+;15B03-Gal4/+*) (*n* = 9). Data are presented as mean values ± SD. **g**, Box plot represents the number of Cut^{+} progenitors in control (*tub-Gal80^{ts}/+; 15B03-Gal4/+*) (*n* = 11) and muscle-perturbed flies (*UAS-rpr,hid/+; tub-Gal80^{ts}/+; 15B03-Gal4/+*) (*n* = 14). Data are presented as median with minima and maxima. 25th–75th percentile (box) and 5th–95th percentile (whiskers). **f,g**, Unpaired two-tailed *t*-test was used for all statistical analyses. At least four biologically independent experiments were performed. No adjustments were made for multiple comparisons. **h-i'**, Expression of *mys:GFP* in trachea. DAPI (blue). **j-k'**, Expression of *Osi15:GFP* in trachea. DAPI (blue). **h-k'**, Scale bars: 30 μm. Source data are provided as a Source Data file.

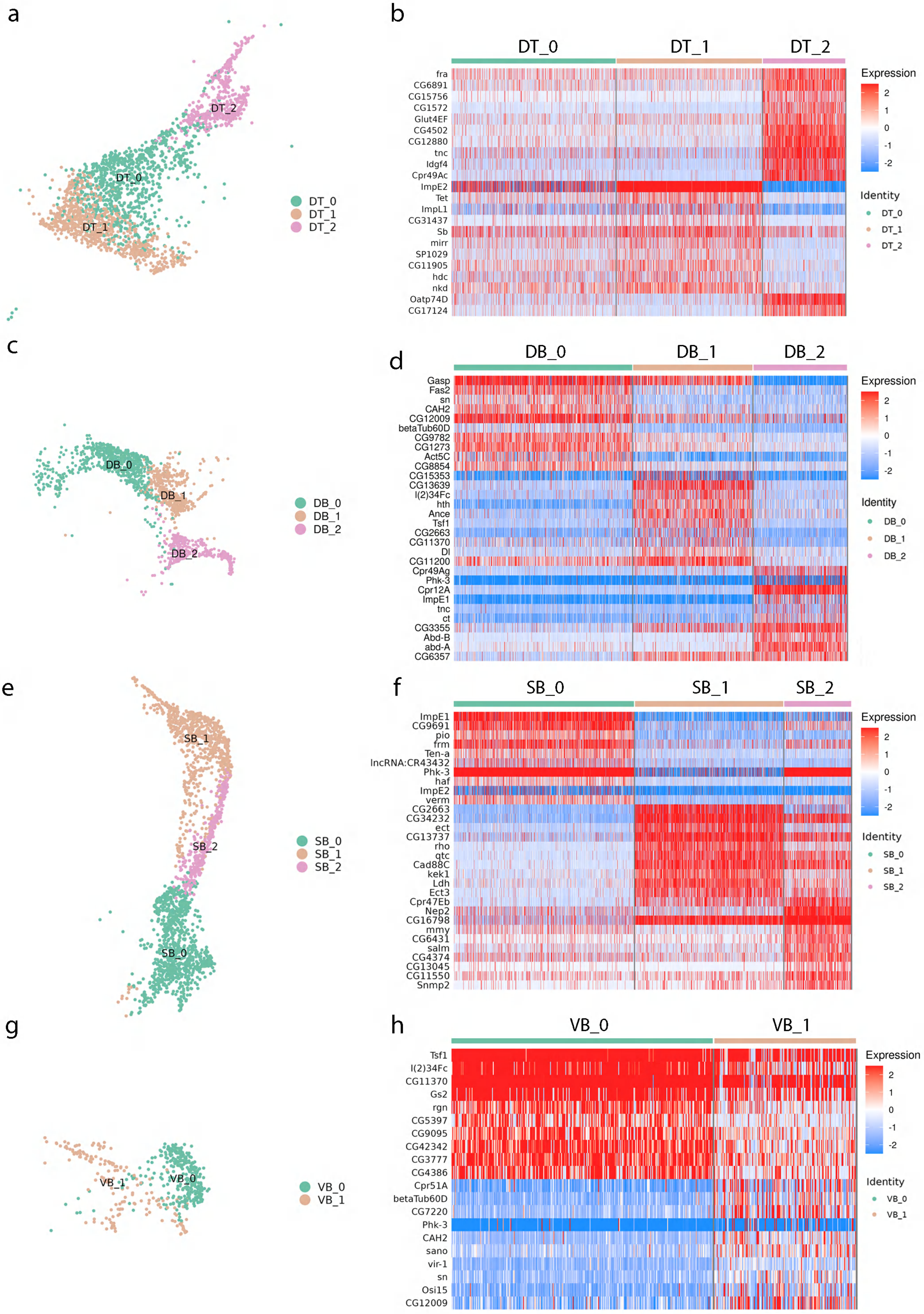
a
DT_0
DT_1
DT_2
b
DT_0 DT_1 DT_2
fra
CG6891
CG15756
CG1572
Glut4EF
CG4502
CG12880
tnc
Idgf4
Cpr49Ac
ImpE2
Tet
ImpL1
CG31437
Sb
mirr
SP1029
CG11905
hdc
nkd
Oatp74D
CG17124
Expression
2
1
0
-1
-2
Identity
c
DB_0
DB_1
DB_2
d
Gasp
Fas2
sn
CAH2
CG12009
betaTub60D
CG9782
CG1273
Act5C
CG8854
CG15353
CG13639
l(2)34Fc
hth
Ance
Tsf1
CG2663
CG11370
Dl
CG11200
Cpr49Ag
Phk-3
Cpr12A
ImpE1
tnc
ct
CG3355
Abd-B
abd-A
CG6357
e
SB_0
SB_1
SB_2
f
ImpE1
CG9691
pio
frm
Ten-a
lncRNA:CR43432
Phk-3
haf
ImpE2
verm
CG2663
CG34232
ect
CG13737
rho
qtc
Cad88C
kek1
Ldh
Ect3
Cpr47Eb
Nep2
CG16798
mmy
CG6431
salm
CG4374
CG13045
CG11550
Snmp2
g
VB_0
VB_1
h
Tsf1
l(2)34Fc
CG11370
Gs2
rgn
CG5397
CG9095
CG42342
CG3777
CG4386
Cpr51A
betaTub60D
CG7220
Phk-3
CAH2
sano
vir-1
sn
Osi15
CG12009

Supplementary Figure 5. Subclustering analysis of DT, DB, SB and VB. a,**c**,**e**,**g**, UMAP plot representing subclusters of DT (**a**), DB (**c**), SB (**e**) and VB (**g**) populations. **b**,**d**,**f**,**h**, Heatmap showing the expression of the unique genes for each subcluster in DT (**b**), DB (**d**), SB (**f**) and VB (**h**).

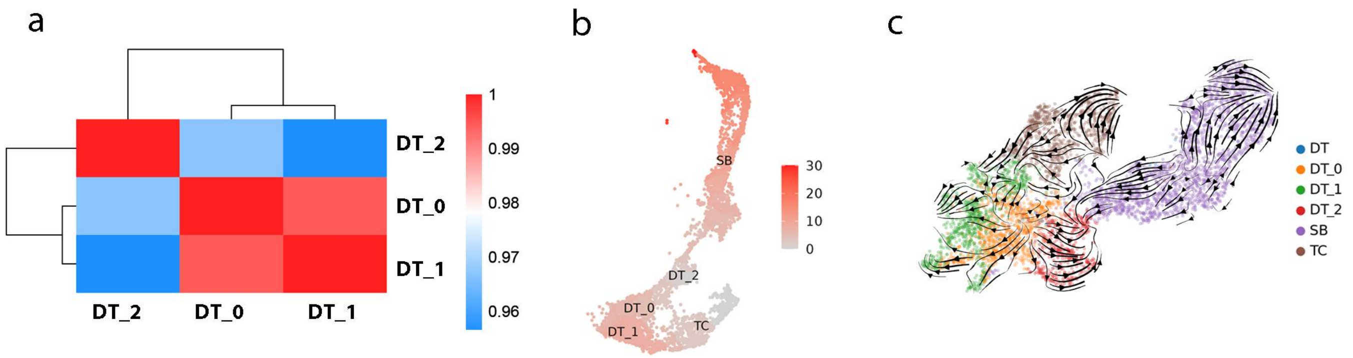
a
DT_2
DT_0
DT_1
DT_2
DT_0
DT_1
1
0.99
0.98
0.97
0.96
b
SB
DT_2
DT_0
DT_1
TC
30
20
10
0
c
DT
DT_0
DT_1
DT_2
SB
TC

Supplementary Figure 6. Diversification of DT population. a, Correlation heatmap of DT subclusters. **b**, State of differentiation of DT, SB and TC cells. **c**, Cell maturation map of DT, SB and TC cells estimated by RNA velocity.

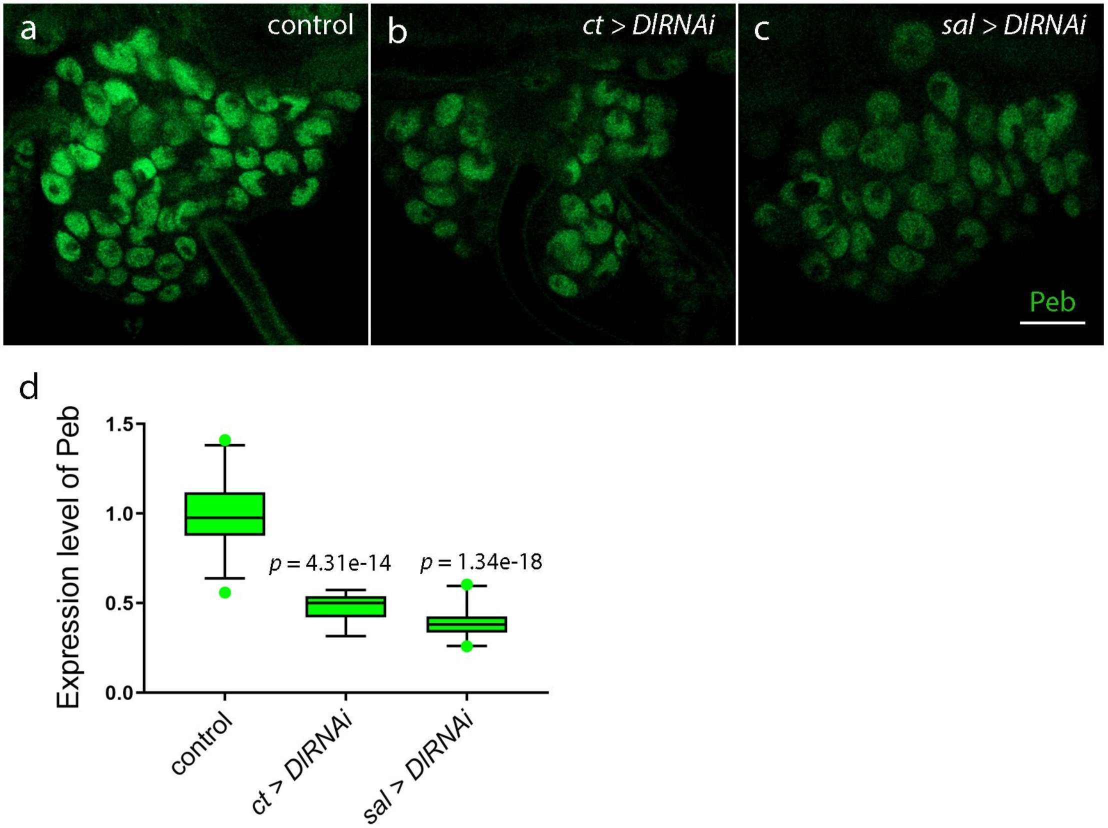
a
control
b
ct > DlRNAi
c
sal > DlRNAi
Peb
d
Expression level of Peb
1.5
1.0
0.5
0.0
p = 4.31e-14
p = 1.34e-18
control
ct > DlRNAi
sal > DlRNAi

Supplementary Figure 7. Progenitors received Notch signals generated by multiple branches. a-c, Staining PC of control (**a**) or *DlRNAi* under the control of *ct*-Gal4 (**b**) or *sal*-Gal4 with α-Peb antibodies (**c**). Scale bars: 20 μm (**a**-**c**). **d**, Box plot represents the expression levels of Peb in control (*UAS-DlRNAi/+*) (n = 32), *cut-Gal4>DlRNAi* flies (*ct-Gal4/+*; *UAS-DlRNAi/+*) (n = 17) or *sal-Gal4>DlRNAi* flies (*UAS-DlRNAi/+*; *sal-Gal4/+*) (n = 21). Data are presented as median with minima and maxima. 25th–75th percentile (box) and 5th–95th percentile (whiskers) as well as outliers are indicated in the box plots. More than six biologically independent experiments were performed. Unpaired two-tailed *t*-test was used for all statistical analyses. No adjustments were made for multiple comparisons. Source data are provided as a Source Data file.

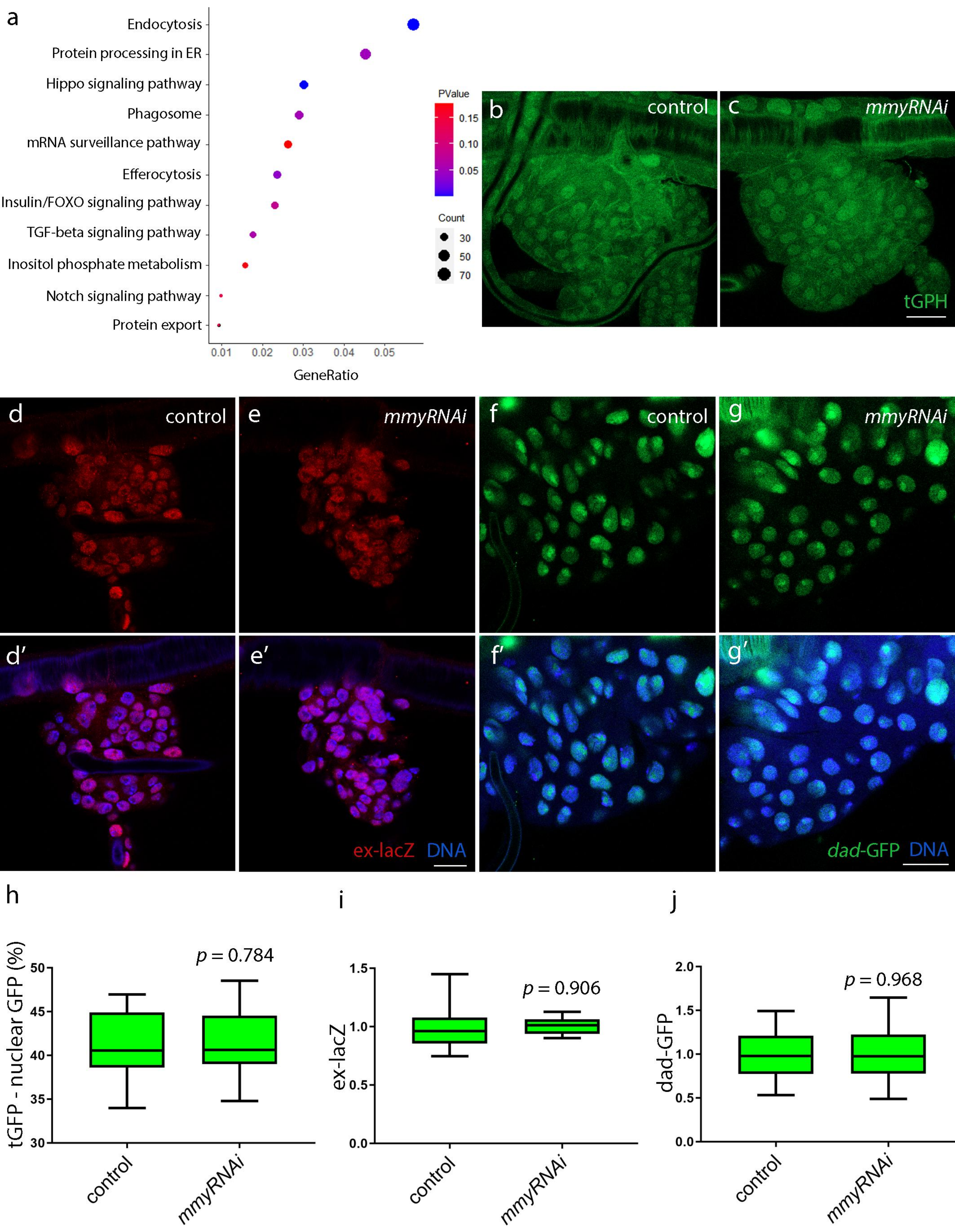

a
Endocytosis
Protein processing in ER
Hippo signaling pathway
Phagosome
mRNA surveillance pathway
Efferocytosis
Insulin/FOXO signaling pathway
TGF-beta signaling pathway
Inositol phosphate metabolism
Notch signaling pathway
Protein export
0.01 0.02 0.03 0.04 0.05
GeneRatio
PValue
0.15
0.10
0.05
Count
30
50
70
b control
c mmyRNAi
tGPH
d control
e mmyRNAi
f control
g mmyRNAi
d′
e′
ex-lacZ DNA
f′
g′
dad-GFP DNA
h
i
j
tGFP - nuclear GFP (%)
p = 0.784
50
45
40
35
30
control
mmyRNAi
ex-lacZ
p = 0.906
1.5
1.0
0.5
0.0
control
mmyRNAi
dad-GFP
p = 0.968
2.0
1.5
1.0
0.5
0.0
control
mmyRNAi

Supplementary Figure 8. Activity of Insulin, Hippo or Dpp/TGF-β signaling in *mmyRNAi* flies. a, GO analysis reveals top functional clusters among the differentially expressed genes (DEGs). **b-g'**, Insulin (**b**,**c**), Hippo (**d**-**e'**) or Dpp/TGF-β (**f-g'**) signaling pathways were not affected by expressing *mmyRNAi*. **h**, Box plot showing the ratio of nuclear GFP in control (*btl-Gal4/+*; *tub-Gal80^{ts}/tGPH*) (*n* = 11) and *mmyRNAi* flies (*btl-Gal4/UAS-mmyRNAi*; *tub-Gal80^{ts}/tGPH*) (*n* = 14). **i**, Box plot represents the expression of ex-lacZ in control (*ex-lacZ/+*; *btl-Gal4/tub-Gal80^{ts}*) (*n* = 14) and *mmyRNAi* flies (*ex-lacZ/UAS-mmyRNAi*; *btl-Gal4/tub-Gal80^{ts}*) (*n* = 13). **j**, Box plot showing the expression levels of *dad*-GFP in control (*btl-Gal4/+*; *dad-GFP/tub-Gal80^{ts}*) (*n* = 14) and *mmyRNAi* flies (*btl-Gal4/UAS-mmyRNAi*; *dad-GFP/tub-Gal80^{ts}*) (*n* = 10). **h-j**, Data are presented as median with minima and maxima. 25th–75th percentile (box) and 5th–95th percentile (whiskers) as well as outliers are indicated in the box plots. More than four biologically independent experiments were performed. Unpaired two-tailed *t*-test was used for all statistical analyses. No adjustments were made for multiple comparisons. Scale bars: 20 μm (**b-g'**). Source data are provided as a Source Data file.

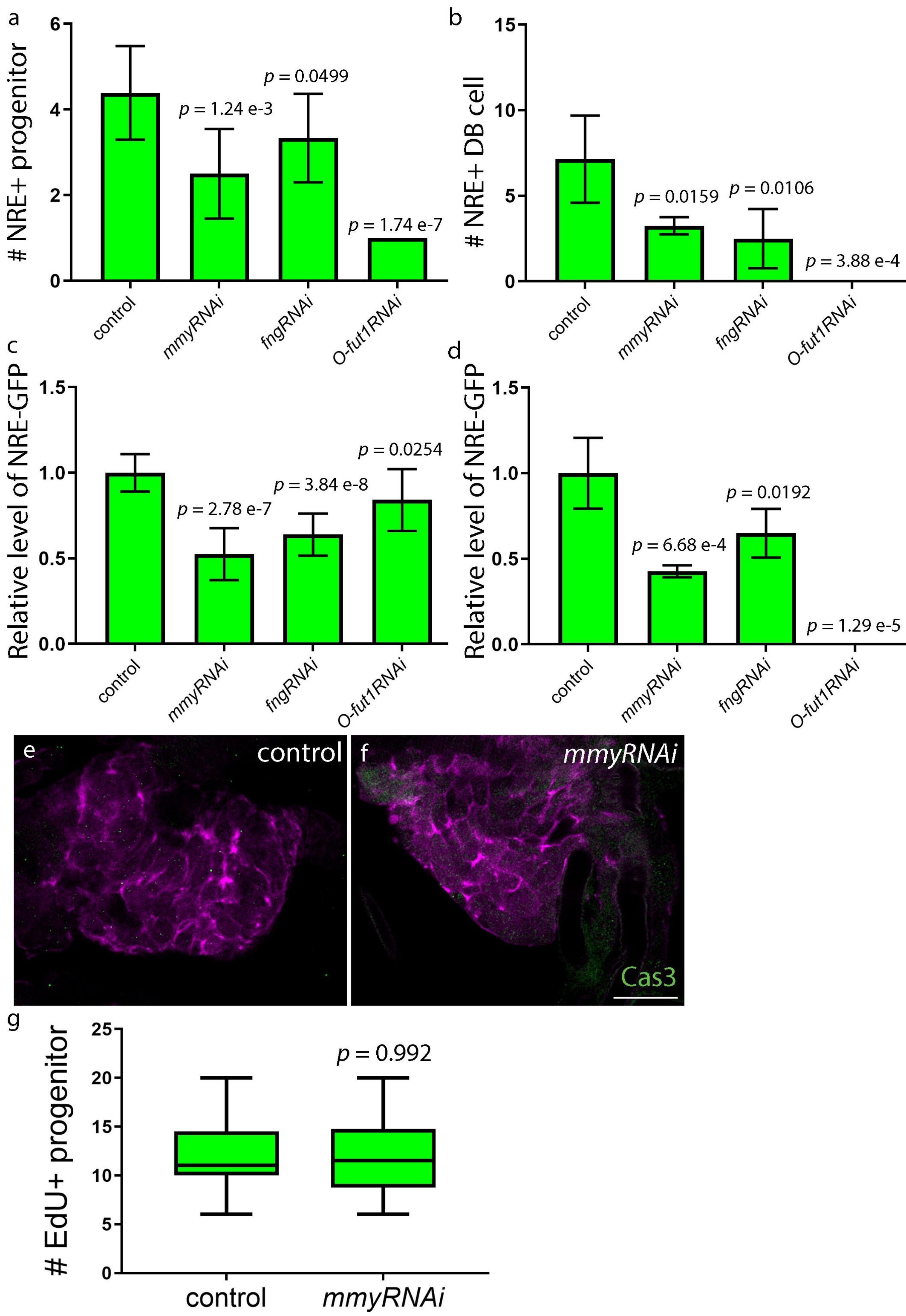
a
NRE+ progenitor
6
4
2
0
p = 1.24 e-3
p = 0.0499
p = 1.74 e-7
control
mmyRNAi
fngRNAi
O-fut1RNAi
b
NRE+ DB cell
15
10
5
0
p = 0.0159
p = 0.0106
p = 3.88 e-4
control
mmyRNAi
fngRNAi
O-fut1RNAi
c
Relative level of NRE-GFP
1.5
1.0
0.5
0.0
p = 2.78 e-7
p = 3.84 e-8
p = 0.0254
control
mmyRNAi
fngRNAi
O-fut1RNAi
d
Relative level of NRE-GFP
1.5
1.0
0.5
0.0
p = 6.68 e-4
p = 0.0192
p = 1.29 e-5
control
mmyRNAi
fngRNAi
O-fut1RNAi
e
control
f
mmyRNAi
Cas3
g
EdU+ progenitor
25
20
15
10
5
0
p = 0.992
control
mmyRNAi

Supplementary Figure 9. Quantification of N activity, cell proliferation, cell death in *mmyRNAi* flies. a-d, Bar graph represents the quantification of NRE-GFP reporter in both PC (**a**,**c**) and DB (**b**,**d**) cells of control (*Su(H)-NRE-GFP/tub-Gal80^{ts}*) (*n* = 18), *mmyRNAi* flies (*btl-Gal4*/*UAS-mmyRNAi*; *Su(H)-NRE-GFP/tub-Gal80^{ts}*) (*n* = 6), *fngRNAi* flies (*btl-Gal4*/*UAS-fngRNAi*; *Su(H)-NRE-GFP/tub-Gal80^{ts}*) (*n* = 6) and *O-fut1RNAi* flies (*btl-Gal4*/*UAS-O-fut1RNAi*; *Su(H)-NRE-GFP/tub-Gal80^{ts}*) (*n* = 6). Data are presented as mean values ± SD. **e**,**f**, Staining tracheal progenitors with cleaved Caspase3 antibody in control (**e**) or *mmyRNAi* (**f**) flies. Scale bars: 30 μm (**e**,**f**). **g**, Box plot showing the number of EdU incorporation in the Tr4 tracheal progenitors of control (*btl-Gal4/+*; *tub-Gal80^{ts}/+*) (n = 17) and *mmyRNAi* (*btl-Gal4/UAS-mmyRNAi*; *tub-Gal80^{ts}/+*) (n = 18) flies. Data are presented as median with minima and maxima. 25th–75th percentile (box) and 5th–95th percentile (whiskers) as well as outliers are indicated in the box plots. **a**-**d**, **g**, More than three biologically independent experiments were performed. Unpaired two-tailed *t*-test was used for all statistical analyses. No adjustments were made for multiple comparisons. Source data are provided as a Source Data file.

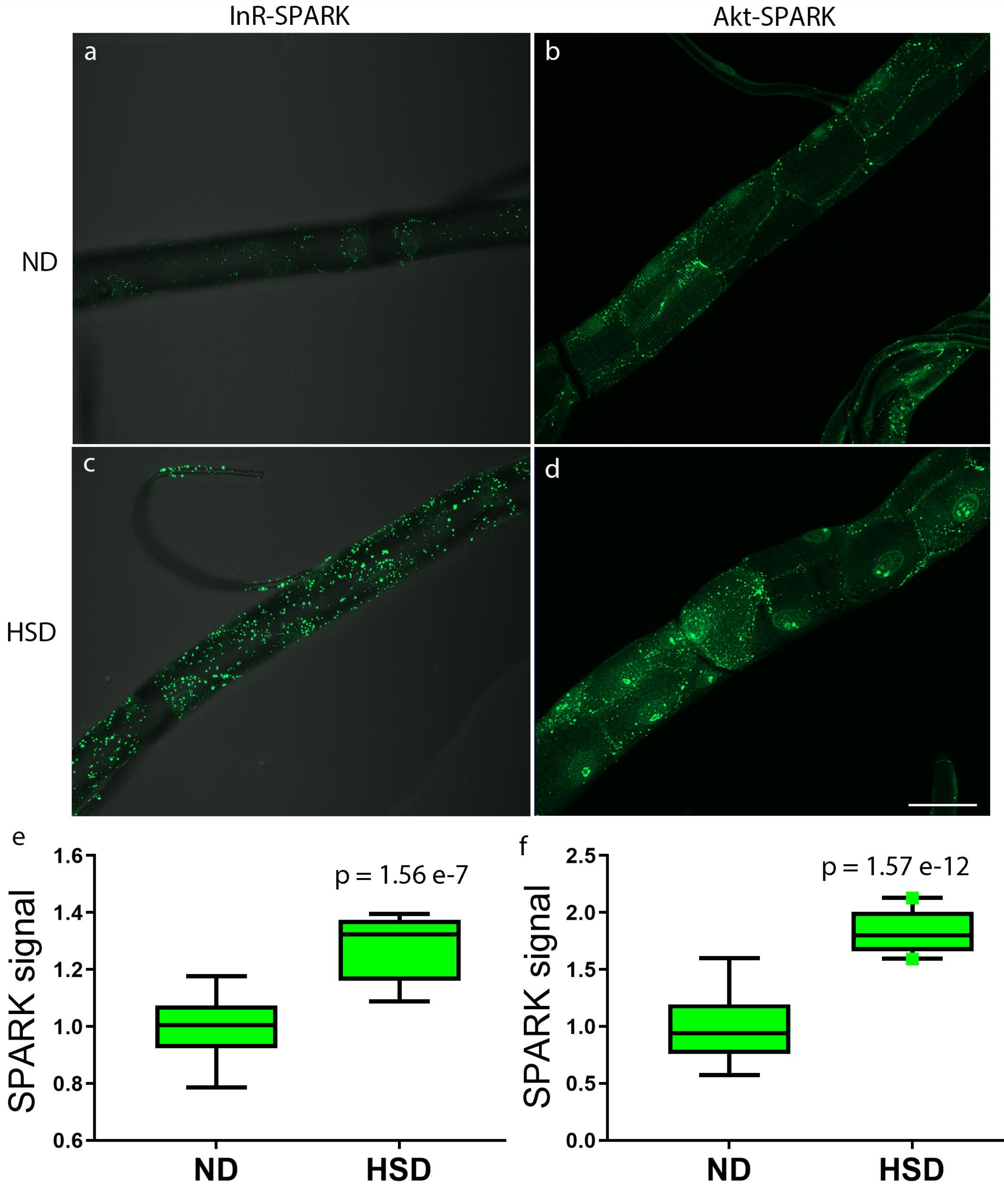

InR-SPARK
Akt-SPARK
a
b
c
d
ND
HSD
e
f
p = 1.56 e-7
p = 1.57 e-12
SPARK signal
1.6
1.4
1.2
1.0
0.8
0.6
2.5
2.0
1.5
1.0
0.5
0.0
ND
HSD

Supplementary Figure 10. Activation of insulin signaling in trachea cells under high sugar condition. a-d, Expression of InR-SPARK (*btl-Gal4/+; UAS-InR-SPARK/+*) (**a**,**c**) and Akt-SPARK (*btl-Gal4/+; UAS-Akt-SPARK/+*) (**b**,**d**) reporters under ND (**a**,**b**) or HSD (**c**,**d**). The number and size of GFP droplets represent signal activation. Scar bars: 50 μm. **e**, Box plot represents the relative signals of InR-SPARK under ND (n = 15) or HSD (n = 19). **f**, Box plot showing the relative signals of Akt-SPARK under ND (n = 16) or HSD (n = 21). **e**,**f**, Data are presented as median with minima and maxima. 25th–75th percentile (box) and 5th–95th percentile (whiskers) as well as outliers are indicated in the box plots. More than three biologically independent experiments were performed. Unpaired two-tailed *t*-test was used for all statistical analyses. No adjustments were made for multiple comparisons. Source data are provided as a Source Data file.

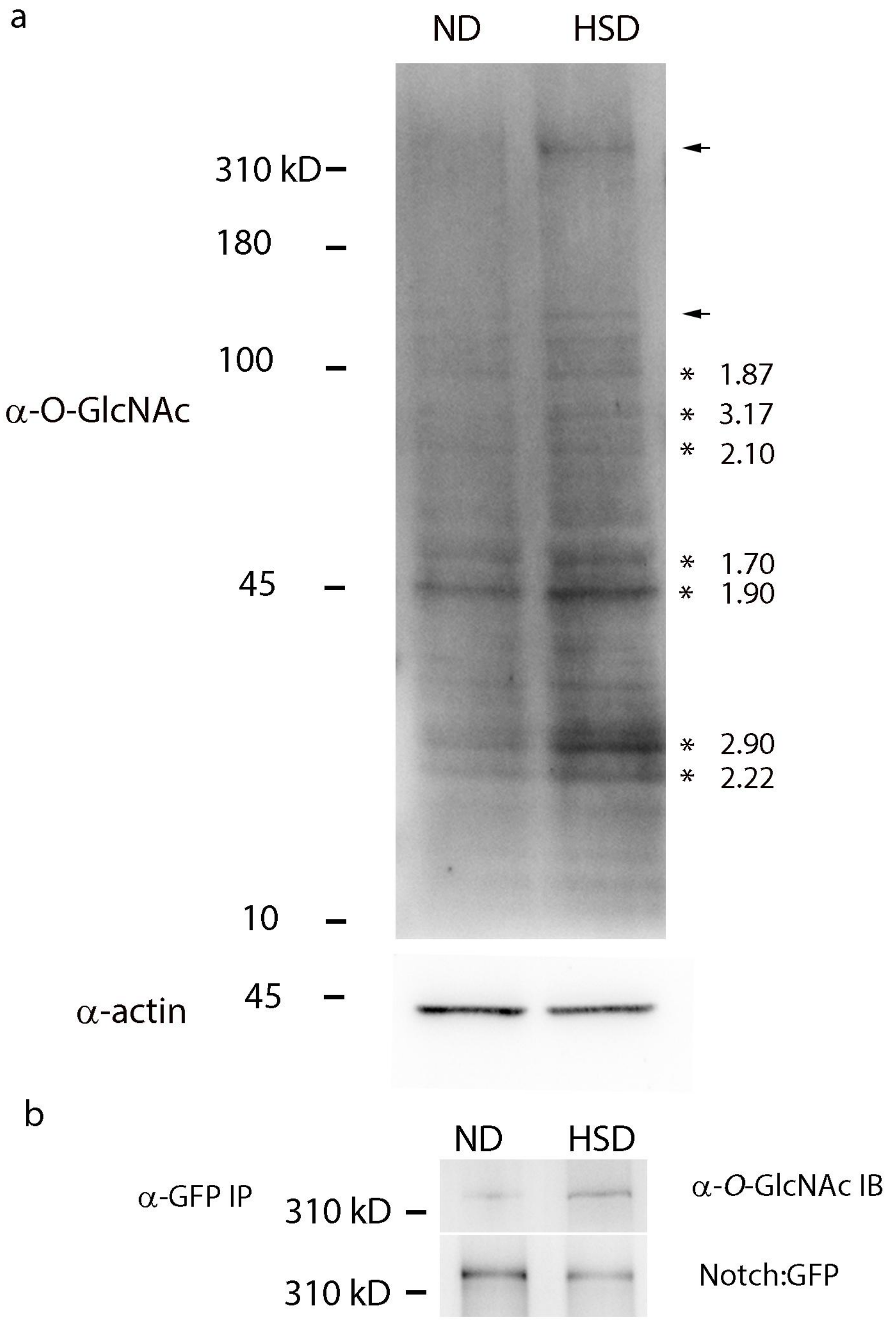
a
ND
HSD
310 kD
180
100
45
10
α-O-GlcNAc
* 1.87
* 3.17
* 2.10
* 1.70
* 1.90
* 2.90
* 2.22
α-actin
45
b
ND
HSD
α-GFP IP
310 kD
α-O-GlcNAc IB
310 kD
Notch:GFP

Supplementary Figure 11. *O*-GlcNAcylated proteins from *Drosophila* trachea in ND or HSD. a, Western blot analysis of protein extracts with antibodies against *O*-GlcNAc or Actin. Arrows point to the bands that were detected in HSD group but not in ND. Asterisks indicate protein bands that were enhanced in HSD. The fold change relative to ND group was normalized to actin. **b**, *O*-GlcNAcylated Notch protein in ND and HSD flies. Notch:GFP protein were precipitated by α-GFP antibodies. The level of *O*-GlcNAc associated Notch was measured by Western blot with α-*O*-GlcNAc. Source data are provided as a Source Data file.

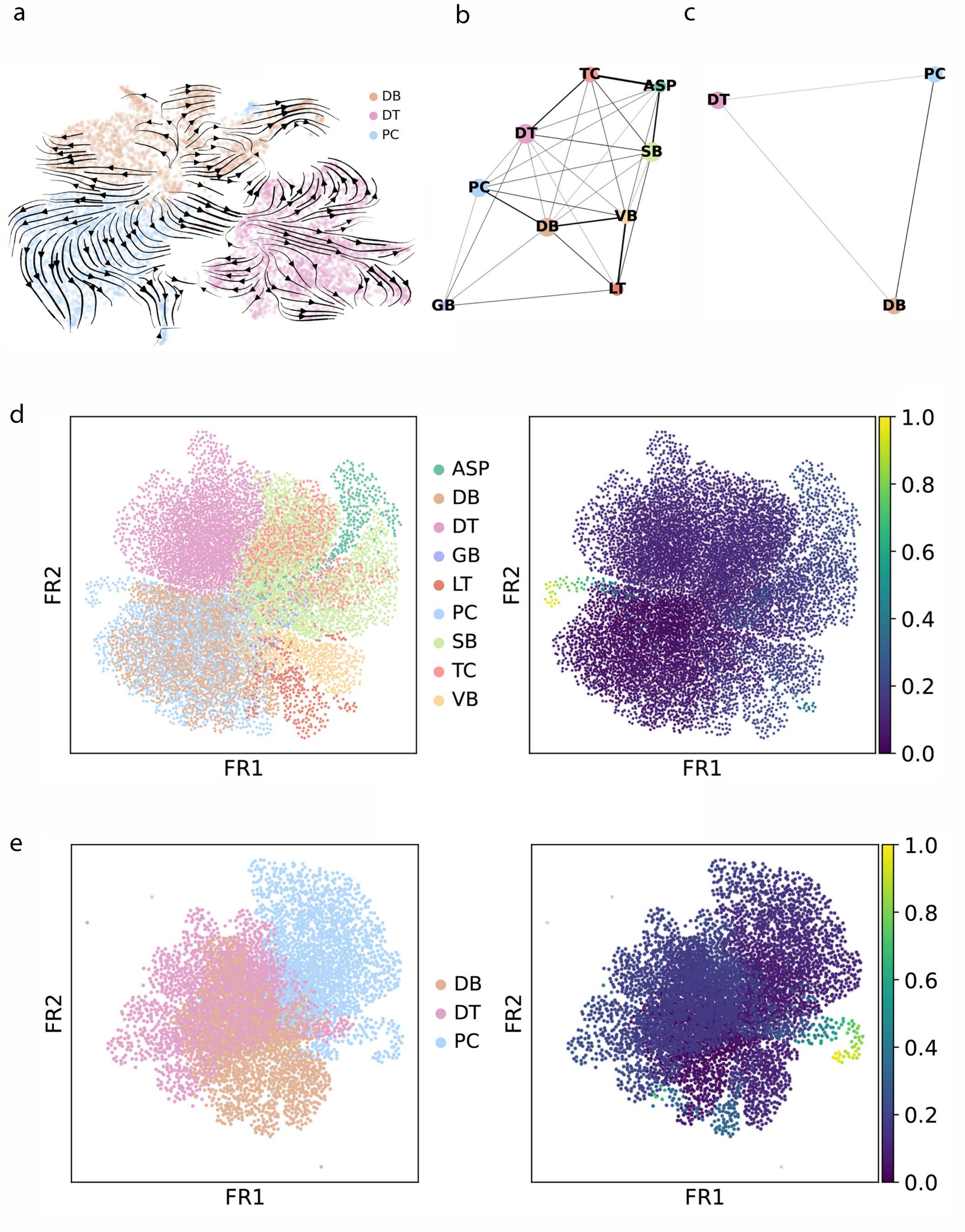
a
DB
DT
PC
b
TC
ASP
DT
SB
PC
VB
DB
LT
GB
c
DT
PC
DB
d
ASP
DB
DT
GB
LT
PC
SB
TC
VB
FR1
FR2
1.0
0.8
0.6
0.4
0.2
0.0
e
DB
DT
PC
FR1
FR2
1.0
0.8
0.6
0.4
0.2
0.0

Supplementary Figure 12. Trajectory inference of tracheoblasts. a, Diagram showing cell maturation between PC, DB and DT as estimated by RNA velocity. **b**, Network diagram showing cell conversion of tracheoblasts. **c**, Cell differentiation between PC, DB and DT cells. **d**, Cell maturation of tracheoblasts inferred by PAGA. **e**, Differentiation status of PC, DB and DT cells.

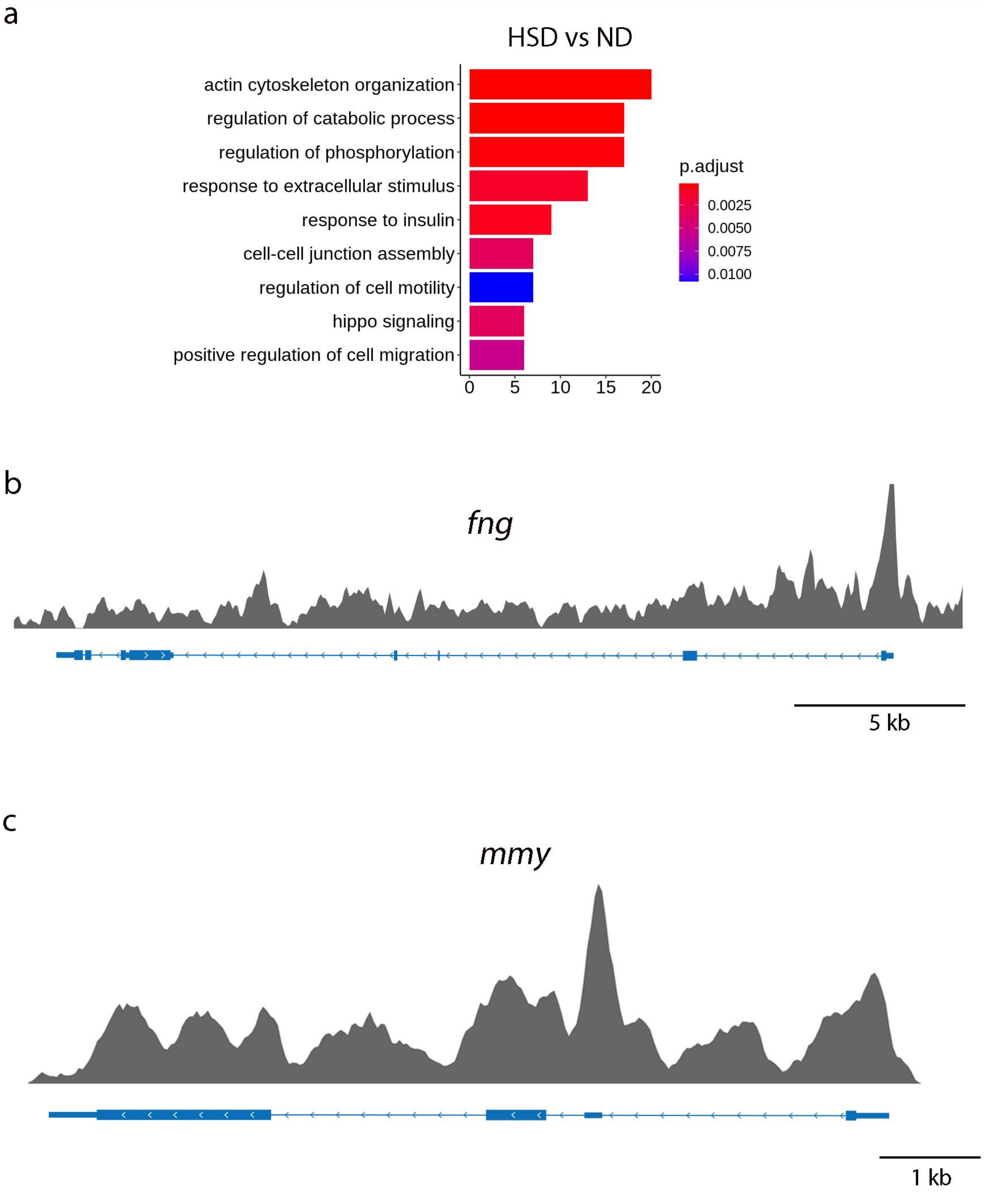
a
HSD vs ND
actin cytoskeleton organization
regulation of catabolic process
regulation of phosphorylation
response to extracellular stimulus
response to insulin
cell-cell junction assembly
regulation of cell motility
hippo signaling
positive regulation of cell migration
0
5
10
15
20
p.adjust
0.0025
0.0050
0.0075
0.0100
b
fng
5 kb
c
mmy
1 kb

Supplementary Figure 13. Targets of Yki signaling participate in glycosylation in *Drosophila* trachea. a, Enriched functional clusters in tracheal progenitors between ND and HSD groups. HSD affects Hippo-Yki signaling pathway in progenitors. **b**,**c**, ChIP-seq peaks at loci of *fng* (**b**) and *mmy* (**c**). Scale bars: 5kb (**b**), 1kb (**c**).

.

Supplementary Table 1

Reagent or Resource	Source	Identifier
**Experimental Models: Organisms/Strains**		
D. melanogaster. btl-Gal4	[7]	
D. melanogaster. btl-RFP-moe	[32]	
D. melanogaster. kni-Gal4	[83]	
D. melanogaster. esg-Gal4	[43]	
D. melanogaster. ct-Gal4	[19]	
D. melanogaster. UAS-mmyRNAi	Vienna Drosophila RNAi Center	VDRC: 105829
D. melanogaster. UAS-bru2RNAi	Bloomington Drosophila Stock Center	BDSC: 50631
D. melanogaster. UAS-sal	Bloomington Drosophila Stock Center	BDSC: 29715
D. melanogaster. sal-Gal4	Tsinghua Stock Center	TB00012
D.melanogaster. Su(H)-NRE-GFP	Bloomington Drosophila Stock Center	BDSC: 30728
D.melanogaster. bs-GFP	Bloomington Drosophila Stock Center	BDSC: 93568
D. melanogaster. UAS-Tom	Bloomington Drosophila Stock Center	BDSC: 26685
D. melanogaster. G-trace	Bloomington Drosophila Stock Center	BDSC:28280
D. melanogaster. UAS-expRNAi	Tsinghua Stock Center	THU1661
D. melanogaster. UAS-ctRNAi	Tsinghua Stock Center	THU5930
D. melanogaster. UAS-kniRNAi	Tsinghua Stock Center	THU3692
D. melanogaster. UAS-mirrRNAi	Tsinghua Stock Center	THU2284
D. melanogaster. UAS-wunRNAi	Tsinghua Stock Center	THU0875
D. melanogaster. UAS-fngRNAi	Tsinghua Stock Center	TH201500423.S
D. melanogaster. UAS-DlRNAi	Vienna Drosophila RNAi Center	VDRC: 27187
D. melanogaster. ex-lacZ	[84]	
D. melanogaster. dad-GFP	[85]	
D. melanogaster. UAS-O-fut1RNAi	Tsinghua Stock Center	TH201501129.S
D. melanogaster. UAS- $\textbf{\textit{Notch}}^{DN}$	[43]	

D. melanogaster. mys:GFP	Vienna Drosophila RNAi Center	VDRC: 318282
D. melanogaster. Osi15:GFP	Bloomington Drosophila Stock Center	BDSC:35881
**Antibody/Kit**		
**anti-β-galactosidase**	Developmental Studies Hybridoma Bank	40-1a
**anti-Peb**	Developmental Studies Hybridoma Bank	1G9
**anti-Cut**	Developmental Studies Hybridoma Bank	2B10
**anti-Delta**	Developmental Studies Hybridoma Bank	C594.9B
**anti-Serp**	[86]	
**anti-kni**	[19]	
**anti-Sal**	[19]	
**anti-GFP**	Invitrogen	#A11122
**HRP-conjugated α-rabbit**	Abcam	#ab6721
**HRP-conjugated α-mouse**	ABclonal	#AS094
**Streptavidin magnetic beads**	Thermo Fisher	#88816
**anti-GFP magnetic beads**	Cell Signaling Technology	#67090
**CTD110.6**	Cell Signaling Technology	#9875
**Click-iT® EdU Imaging Kits**	Invitrogen	#C10337
**Click-iT™ *O*-GlcNAc enzymatic labeling system**	Invitrogen	#C33368
**Click-iT™ glycoprotein detection kit**	Invitrogen	#C33372
**SMART-Seq v4**	Takara Bio	
**RNeasy Micro Kit**	Qiagen	#74004
**Software and Algorithms**		
**ZEN (blue edition)**	Carl Zeiss	N/A
**CytoScape (v3.10.0)**	Cytoscape Team	https://cytoscape.org/
**ImageJ**	NIH	https://imagej.net
**GraphPad Prism (v9.5.1)**	GraphPad Software	https://www.graphpad.com/scientific-software/prism/
**Hisat2**		https://ccb.jhu.edu/software/hisat2
**DESeq2**	Bioconductor	https://bioconductor.org/
**Homer**		https://homer.ucsd.edu/homer/
**pyGenomeTracks**		https://pygenometracks.readthedocs.io/
