# Supplementary material for: A single-cell atlas of Drosophila trachea reveals glycosylation-mediated Notch signaling in cell fate specification
Source: Nat Commun. 2024 Mar 6;15:2019. doi: 10.1038/s41467-024-46455-w (PMC10917797; doi:10.1038/s41467-024-46455-w)
Supplement: Supplementary file 3 — Reporting Summary [file 41467_2024_46455_MOESM3_ESM.pdf]

nature portfolio

Corresponding author(s): Hai Huang, Quanyi Zhao, Honggang Wu

Last updated by author(s): Feb 16, 2024

Reporting Summary

Nature Portfolio wishes to improve the reproducibility of the work that we publish. This form provides structure for consistency and transparency in reporting. For further information on Nature Portfolio policies, see our Editorial Policies and the Editorial Policy Checklist.

Statistics

For all statistical analyses, confirm that the following items are present in the figure legend, table legend, main text, or Methods section.

n/a	Confirmed	
☐	☒	The exact sample size (*n*) for each experimental group/condition, given as a discrete number and unit of measurement
☐	☒	A statement on whether measurements were taken from distinct samples or whether the same sample was measured repeatedly
☐	☒	The statistical test(s) used AND whether they are one- or two-sided<br>*Only common tests should be described solely by name; describe more complex techniques in the Methods section.*
☒	☐	A description of all covariates tested
☐	☒	A description of any assumptions or corrections, such as tests of normality and adjustment for multiple comparisons
☐	☒	A full description of the statistical parameters including central tendency (e.g. means) or other basic estimates (e.g. regression coefficient) AND variation (e.g. standard deviation) or associated estimates of uncertainty (e.g. confidence intervals)
☐	☒	For null hypothesis testing, the test statistic (e.g. *F*, *t*, *r*) with confidence intervals, effect sizes, degrees of freedom and *P* value noted<br>*Give P values as exact values whenever suitable.*
☒	☐	For Bayesian analysis, information on the choice of priors and Markov chain Monte Carlo settings
☒	☐	For hierarchical and complex designs, identification of the appropriate level for tests and full reporting of outcomes
☒	☐	Estimates of effect sizes (e.g. Cohen's *d*, Pearson's *r*), indicating how they were calculated

Our web collection on statistics for biologists contains articles on many of the points above.

Software and code

Policy information about availability of computer code

Data collection

- Confocal images were collected using Zen 3.1 (blue edition) software.
- Samples for SMART-seq was sequenced by Hiseq4000 instrument.
- Samples (L3, 0hr APF and 2hr APF) for SMART-seq was sequenced by llumina NovaSeq 6000 instrument.
- Samples for scRNA-seq was sequenced by Illumina NovaSeq 6000 instrument.

Data analysis	- Image analysis was done using ImageJ (v1.53n) https://imagej.nih.gov/ij/.<br>- Statistic analysis was performed by GraphPad Prism 9 (v9.5.1) for Windows https://www.graphpad.com/scientific-software/prism/.<br>- The clean reads of SMART-seq data were mapped to the Drosophila genome sequence using Hisat2 with default parameters.<br>- The number of mapped reads were counted by featureCounts.<br>- Differential gene expression analysis was performed using the DESeq2 package https://bioconductor.org/.<br>- Gene ontology and KEGG pathway enrichment analyses for the differentially expressed genes were conducted using the Database DAVID https://david.ncifcrf.gov/ and PANTHER (v18.0) http://www.pantherdb.org/.<br>- Motif analysis was done with Homer (v4.11) https://homer.ucsd.edu/homer/.<br>- Peaks were called using macs2 callpeak 64 and plotted using pyGenomeTracks (v3.6) https://pygenometracks.readthedocs.io/.<br>- Cellranger was utilized to align reads of scRNA-seq to the reference genome Drosophila melanogaster and to perform batch effect correction and dataset aggregation https://support.10xgenomics.com/single-cell-gene-expression/software/pipelines/latest/what-is-cell-ranger.<br>- The Seurat standard workflow was applied to the filtered and normalized scRNA-seq datasets https://satijalab.org/seurat/.<br>- Pseudotime analysis was performed using R package monocle3 https://cole-trapnell-lab.github.io/monocle3/.<br>- CellChat was used to perform intercellular communication http://www.cellchat.org/.<br>- SCENIC was performed to characterize the enriched regulons https://scenic.aertslab.org/.<br>- All custom scripts are available at https://github.com/Tianfeng-Lu/single-cell-atlas-of-fly-trachea.<br>- The DOI for the Github repository is https://doi.org/10.5281/zenodo.10672045.

For manuscripts utilizing custom algorithms or software that are central to the research but not yet described in published literature, software must be made available to editors and reviewers. We strongly encourage code deposition in a community repository (e.g. GitHub). See the Nature Portfolio guidelines for submitting code & software for further information.

Data

Policy information about availability of data

All manuscripts must include a data availability statement. This statement should provide the following information, where applicable:

- Accession codes, unique identifiers, or web links for publicly available datasets
- A description of any restrictions on data availability
- For clinical datasets or third party data, please ensure that the statement adheres to our policy

All data generated or analysed during this study are included in this published article (and its supplementary information files). The SMART-Seq data (L3, 0hr APF and 2hr APF) generated and analyzed in this study have been deposited in the NCBI database under accession number GSE184856 [https://www.ncbi.nlm.nih.gov/geo/query/acc.cgi?acc=GSE184856]. The single-cell RNA sequencing data and the SMART-Seq data (control and mmyRNAi) generated and analyzed in this study have been deposited in the NCBI database under accession number GSE240777 [https://www.ncbi.nlm.nih.gov/geo/query/acc.cgi?acc=GSE240777]. The databases/datasets used in the study: Drosophila genome (dm6) https://www.ncbi.nlm.nih.gov/assembly/GCF_000001215.4/

Field-specific reporting

Please select the one below that is the best fit for your research. If you are not sure, read the appropriate sections before making your selection.

☒ Life sciences ☐ Behavioural & social sciences ☐ Ecological, evolutionary & environmental sciences

For a reference copy of the document with all sections, see nature.com/documents/nr-reporting-summary-flat.pdf

Life sciences study design

All studies must disclose on these points even when the disclosure is negative.

Sample size	The sample size was determined by our preliminary experiment.<br>- More than 3 white pupae were used for migration experiment, EdU cell proliferation assay, and most immunofluorescence staining. According to our preliminary experiments, 3 is the minimal sample size to obtain convincing and statistically significant conclusions.<br>- To minimize the sample variation between different individuals, clusters of tracheal progenitors were collected from two L3 or pupae that were at the same developmental stage.<br>- Our preliminary results revealed that the sample size needs to be larger than 50, which grants sufficient yield for next generation sequencing.<br>- The size number of GlcNAcylation detection is 150 for each sample. According to our preliminary experiments, smaller sample size may not be sufficient to obtain clear and sharp bands.<br>- The size number of O-GlcNAc detection is 80 for each sample. our preliminary experiments show that smaller sample size may lead to blurry or even undetectable bands.<br>- The size number of Immunoprecipitation is 150 for each sample. The sample size was chosen based on our preliminary experiments to obtain enough protein of interest for the following SDS-PAGE.
Data exclusions	None.
Replication	At least 3 independent biological replicates were performed successfully for most experiments.
Randomization	Animals were allocated to each experimental group randomly for all experiments.
Blinding	Image qualifications were performed blindly. For most experiments, blinding is not possible during sample collection as genotypes of flies

need to be visually distinguished.

Reporting for specific materials, systems and methods

We require information from authors about some types of materials, experimental systems and methods used in many studies. Here, indicate whether each material, system or method listed is relevant to your study. If you are not sure if a list item applies to your research, read the appropriate section before selecting a response.

Materials & experimental systems

n/a	Involved in the study
☐	☒ Antibodies
☒	☐ Eukaryotic cell lines
☒	☐ Palaeontology and archaeology
☐	☒ Animals and other organisms
☒	☐ Human research participants
☒	☐ Clinical data
☒	☐ Dual use research of concern

Methods

n/a	Involved in the study
☒	☐ ChIP-seq
☒	☐ Flow cytometry
☒	☐ MRI-based neuroimaging

Antibodies

Antibodies used

- anti-β-galactosidase (mouse) (1:100) (Developmental Studies Hybridoma Bank, 40-1a)
- anti-Peb (mouse) (1:100) (Developmental Studies Hybridoma Bank, 1G9)
- anti-Cut (mouse) (1:100) (Developmental Studies Hybridoma Bank, 2B10)
- anti-Delta (mouse) (1:100) (Developmental Studies Hybridoma Bank, C594.9B)
- anti-Serp (rabbit) (1:200) (Luschnig, S., 2006)
- anti-Kni (guinea pig) (1:400) (Rao, P. R., 2015)
- anti-Sal (rabbit) (1:200) (Rao, P. R., 2015)
- anti-GFP (rabbit) (1:2000) (Invitrogen, #A-11122)
- anti-O-GlcNAc (mouse) (1:1000) (CST, #9875)
- HRP-conjugated α-mouse (rabbit) (1:5000) (ABclonal, #AS094)
- HRP-conjugated α-rabbit (goat) (1:5000) (Abcam, #ab6721)

Validation

All antibodies used in the study have been validated by the manufacturers or original authors:
- anti-β-galactosidase is validated by Developmental Studies Hybridoma Bank (Yang, Y., 2024)
- anti-Peb is validated by Developmental Studies Hybridoma Bank (Ramesh, P., 2021)
- anti-Cut is validated by Developmental Studies Hybridoma Bank (Zhang, Y., 2023)
- anti-Delta is validated by Developmental Studies Hybridoma Bank (Bellec, K., 2021)
- anti-Serp (Luschnig, S., 2006)
- anti-Kni (Rao, P. R., 2015)
- anti-Sal (Rao, P. R., 2015)
- anti-GFP is validated by Invitrogen (Fankhaenel M., 2023)
- anti-O-GlcNAc s validated by Cell Signaling Technology (Lu P., 2023)

Animals and other organisms

Policy information about studies involving animals; ARRIVE guidelines recommended for reporting animal research

Laboratory animals

The laboratory animal involved in the study is Drosophila melanogaster. Details of the strains used in this study are provided in Supplementary Table 1. L3, 0 hr APF and 2 hr APF of stain btl-RFP-moe were used. L3 of fly btl-Gal4/+; UAS-InR-SPARK/P[B123]-RFP-moe were used. L3 and 1 hr APF of stains mys:GFP and Osi15:GFP were used. 0 hr APF of other stains listed in Supplementary Table 1 were used in the study.

Wild animals

No wild animals were used in the study.

Field-collected samples

No field-collected samples were used in the study.

Ethics oversight

No ethics approval is needed for experiments using Drosophila.

Note that full information on the approval of the study protocol must also be provided in the manuscript.
