# Supplementary figures and images for: A single-cell atlas of Drosophila trachea reveals glycosylation-mediated Notch signaling in cell fate specification
Source: Nat Commun. 2024 Mar 6;15:2019. doi: 10.1038/s41467-024-46455-w (PMC10917797; doi:10.1038/s41467-024-46455-w)

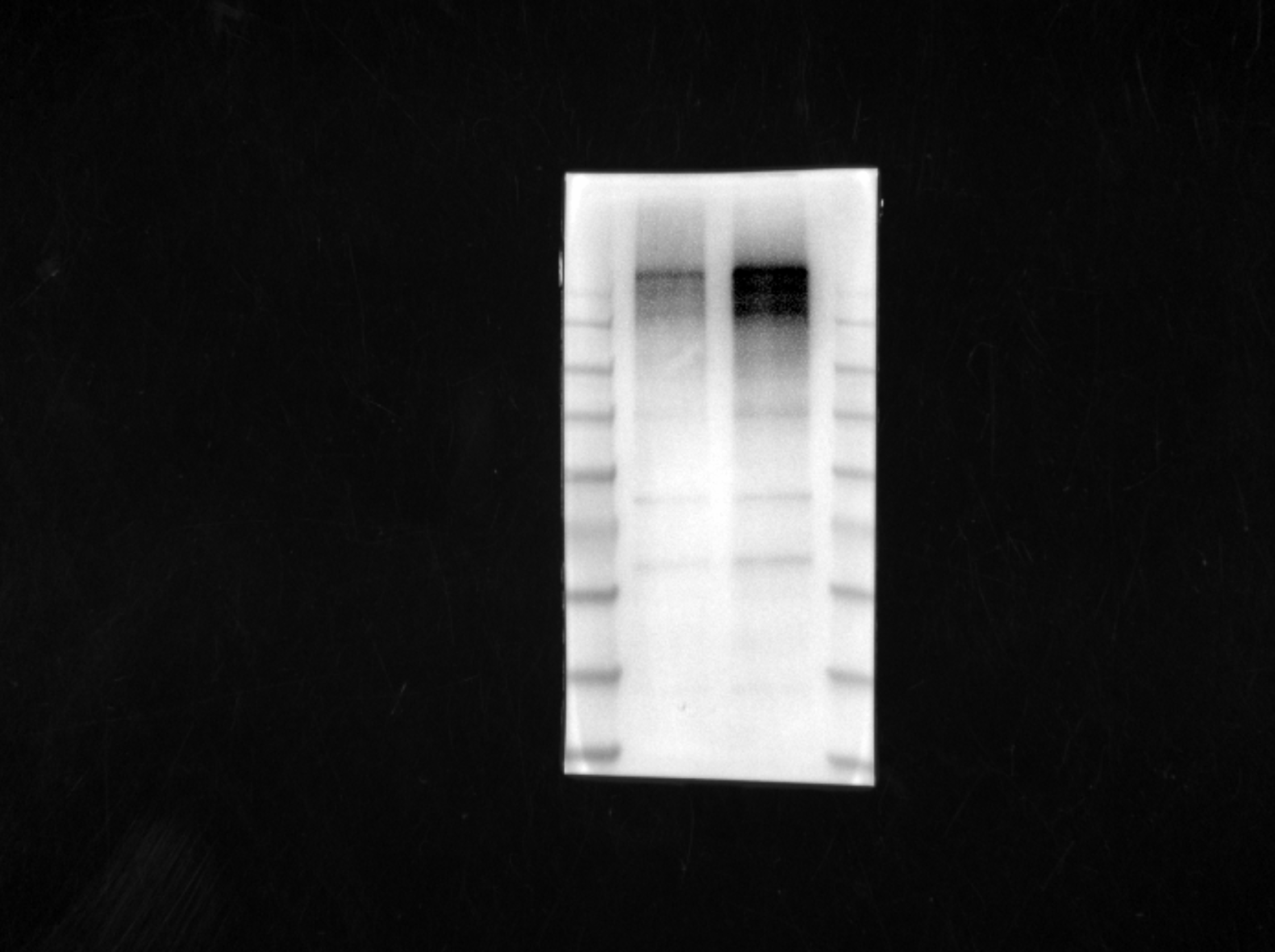

Supplement: Supplementary file 4 — Source data [file 41467_2024_46455_MOESM4_ESM.zip › Source Data/WB for Fig. 6s anti-GFP.tif]

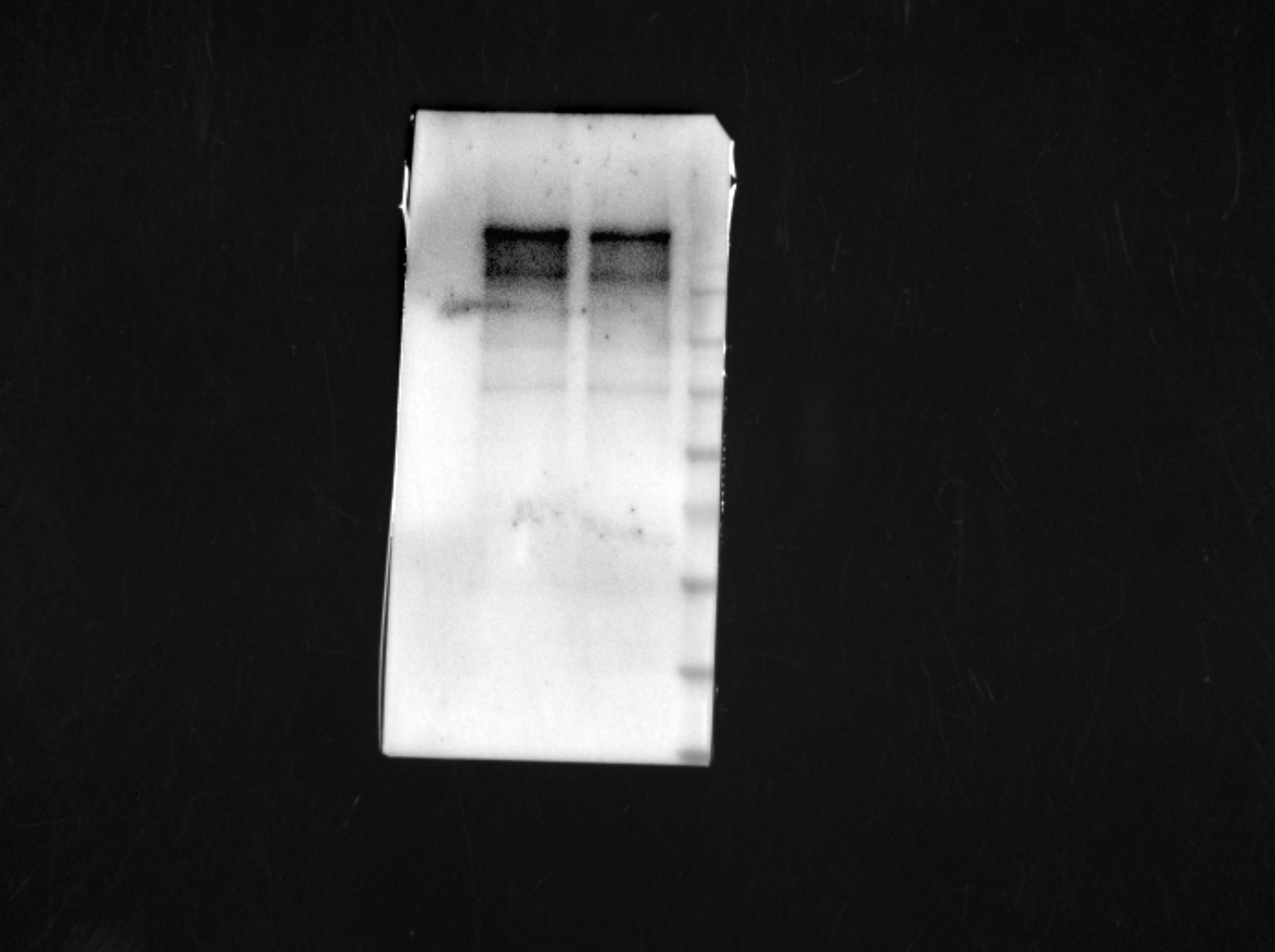

Supplement: Supplementary file 4 — Source data [file 41467_2024_46455_MOESM4_ESM.zip › Source Data/WB for Fig. 6s loading control.tif]

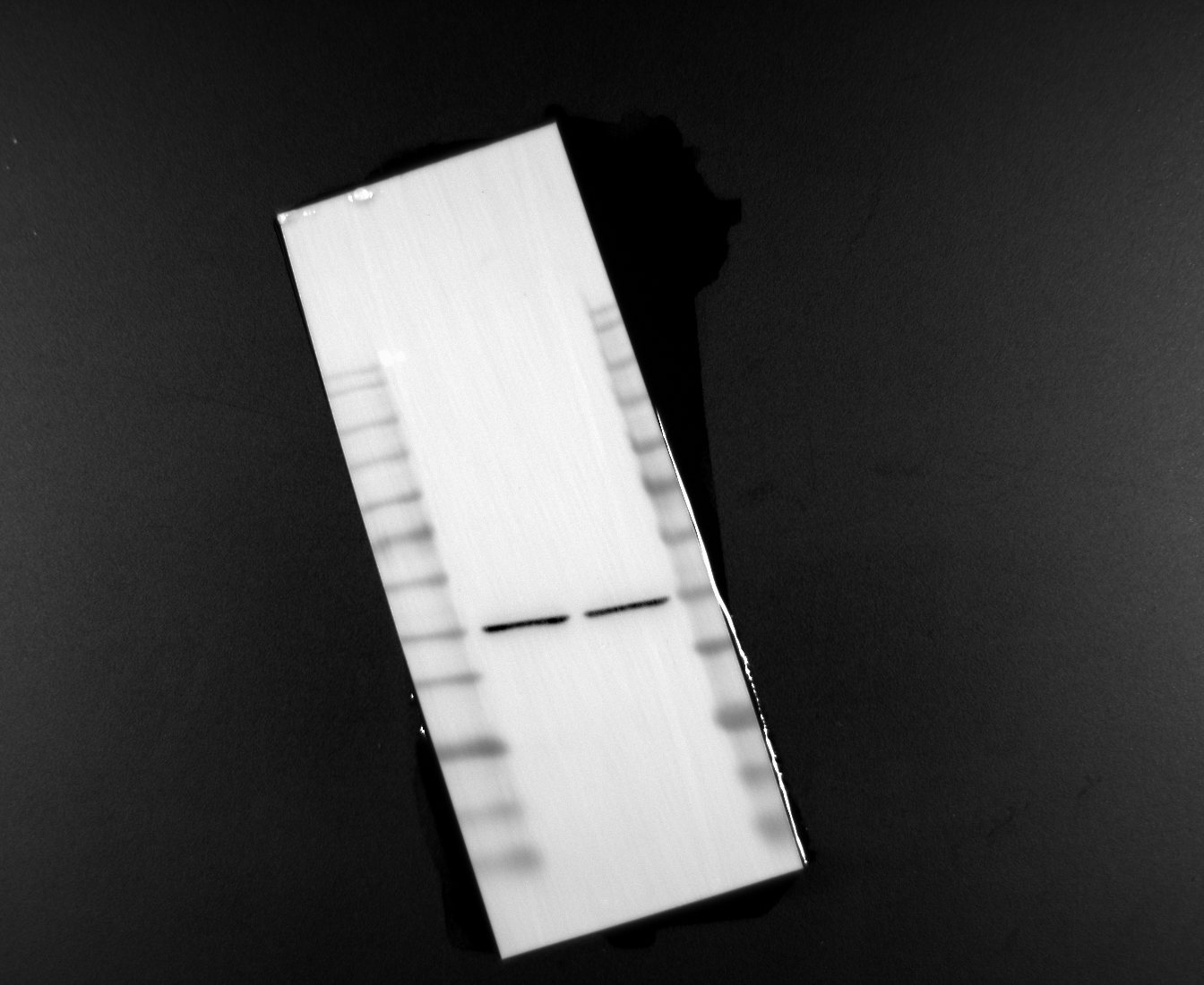

Supplement: Supplementary file 4 — Source data [file 41467_2024_46455_MOESM4_ESM.zip › Source Data/WB for Supplementary Fig. 11a anti-actin.tif]

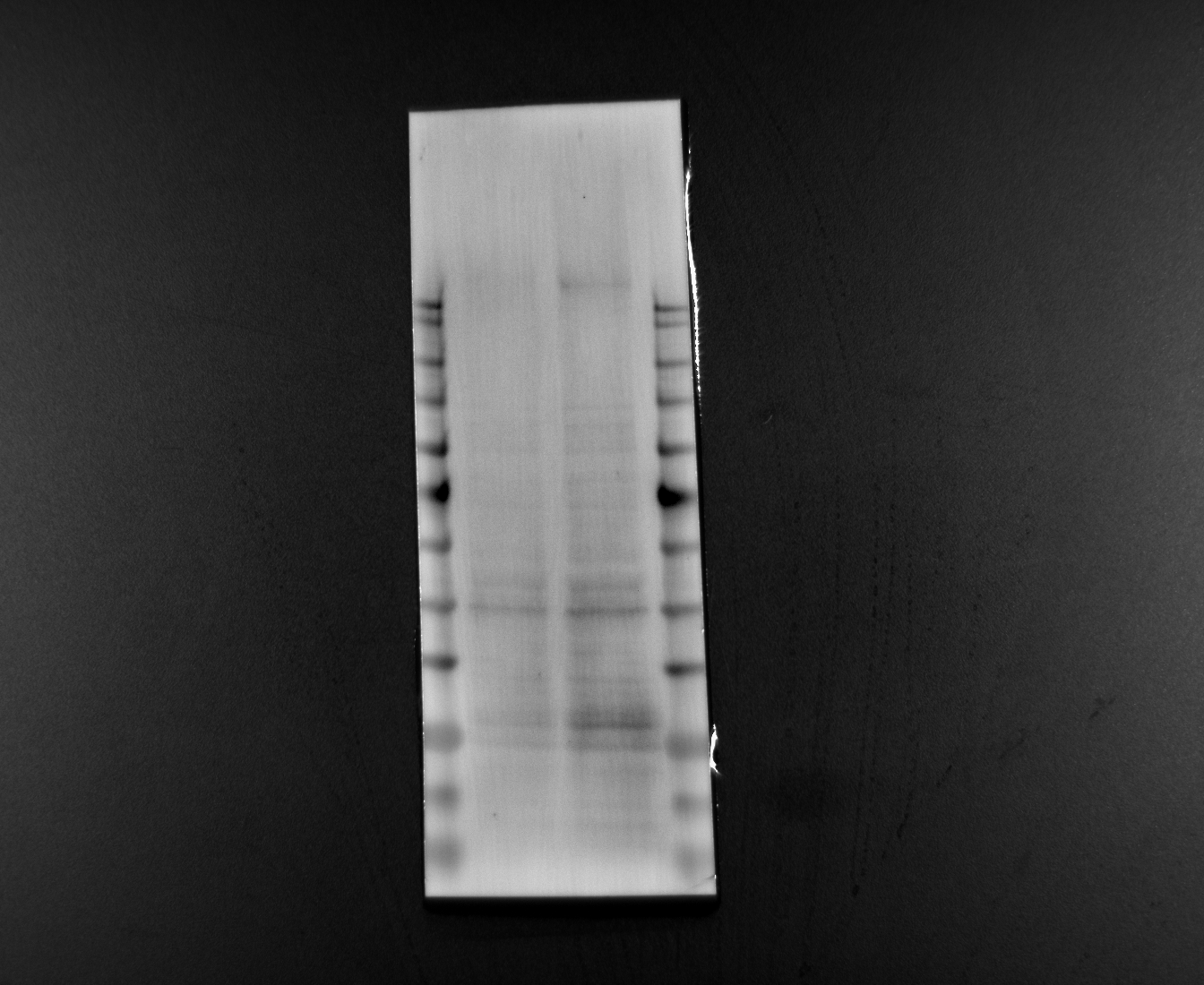

Supplement: Supplementary file 4 — Source data [file 41467_2024_46455_MOESM4_ESM.zip › Source Data/WB for Supplementary Fig. 11a anti-O-GlcNAc.tif]

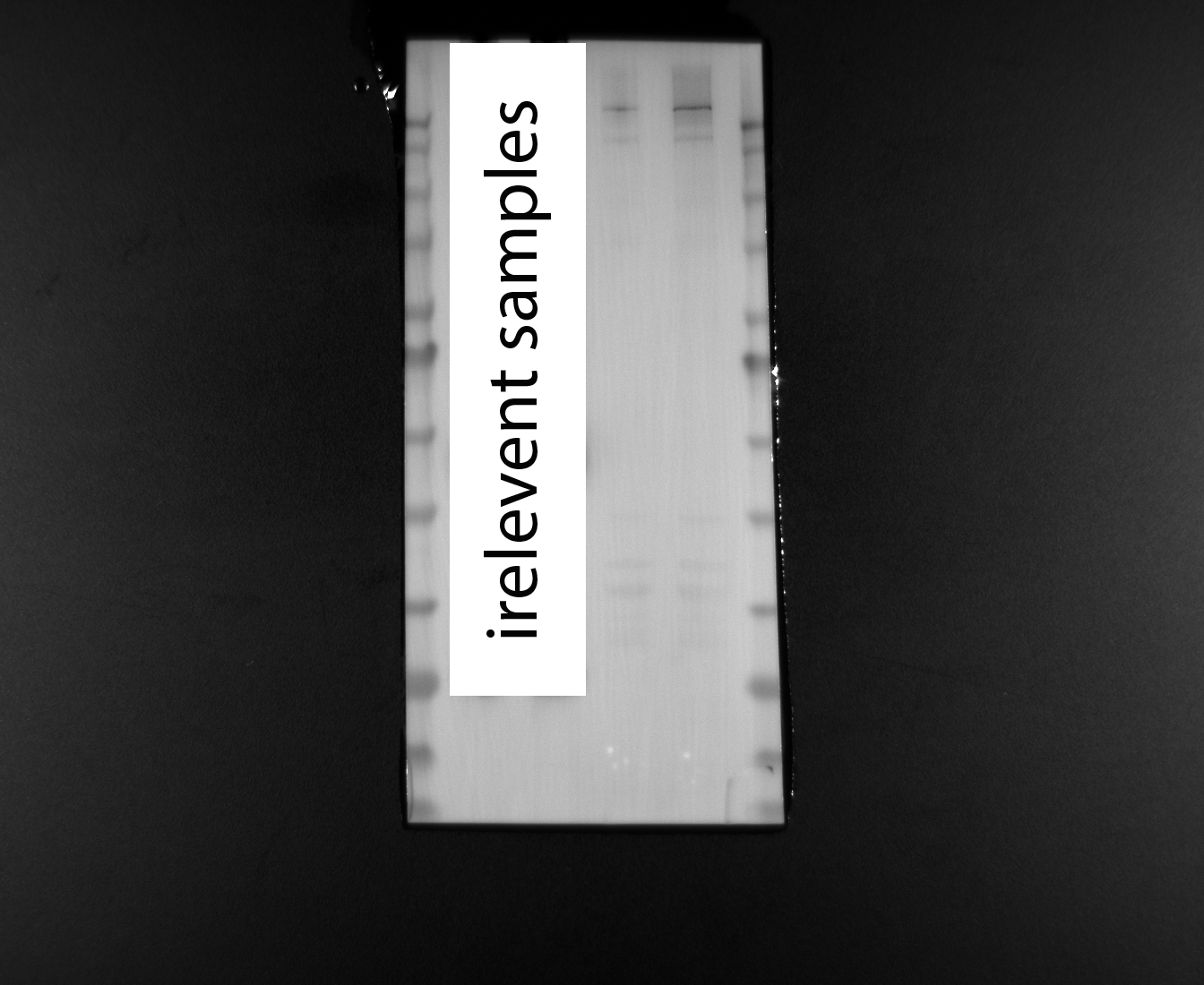

Supplement: Supplementary file 4 — Source data [file 41467_2024_46455_MOESM4_ESM.zip › Source Data/WB for Supplementary Fig. 11b anti-O-GlcNAc.tif]

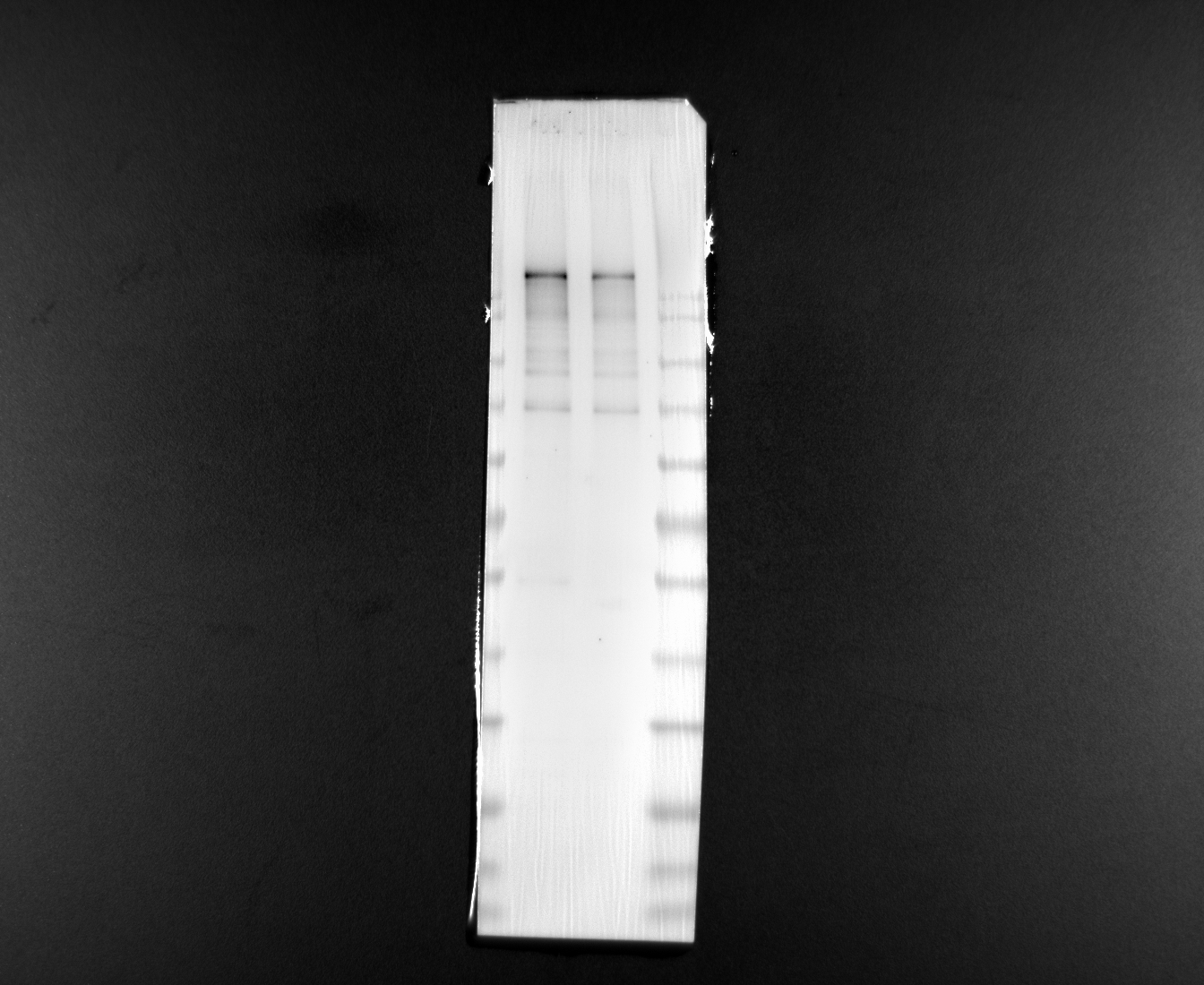

Supplement: Supplementary file 4 — Source data [file 41467_2024_46455_MOESM4_ESM.zip › Source Data/WB for Supplementary Fig. 11b Notch-GFP.tif]

Fig. 6s

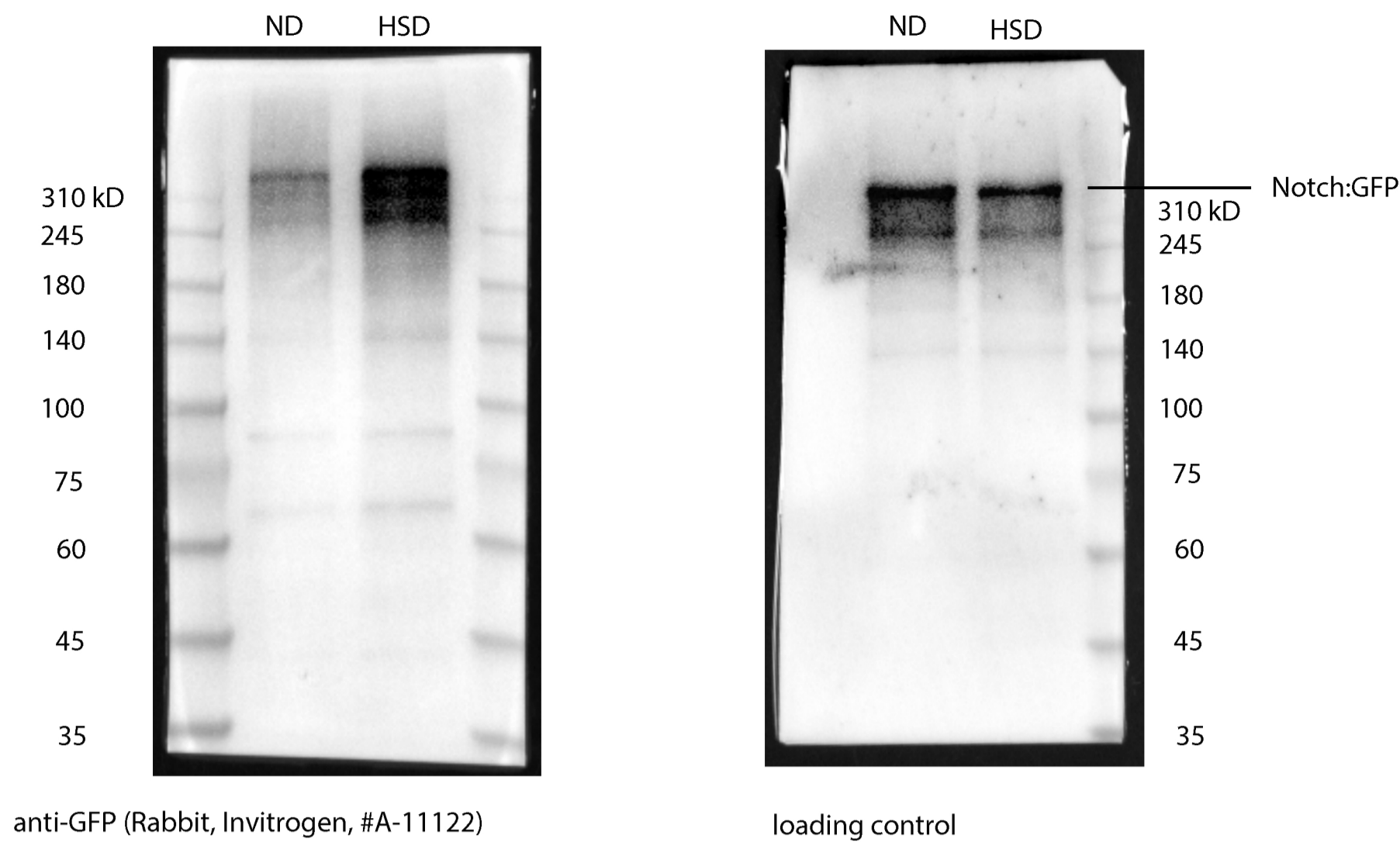

Supplementary Fig. 11a

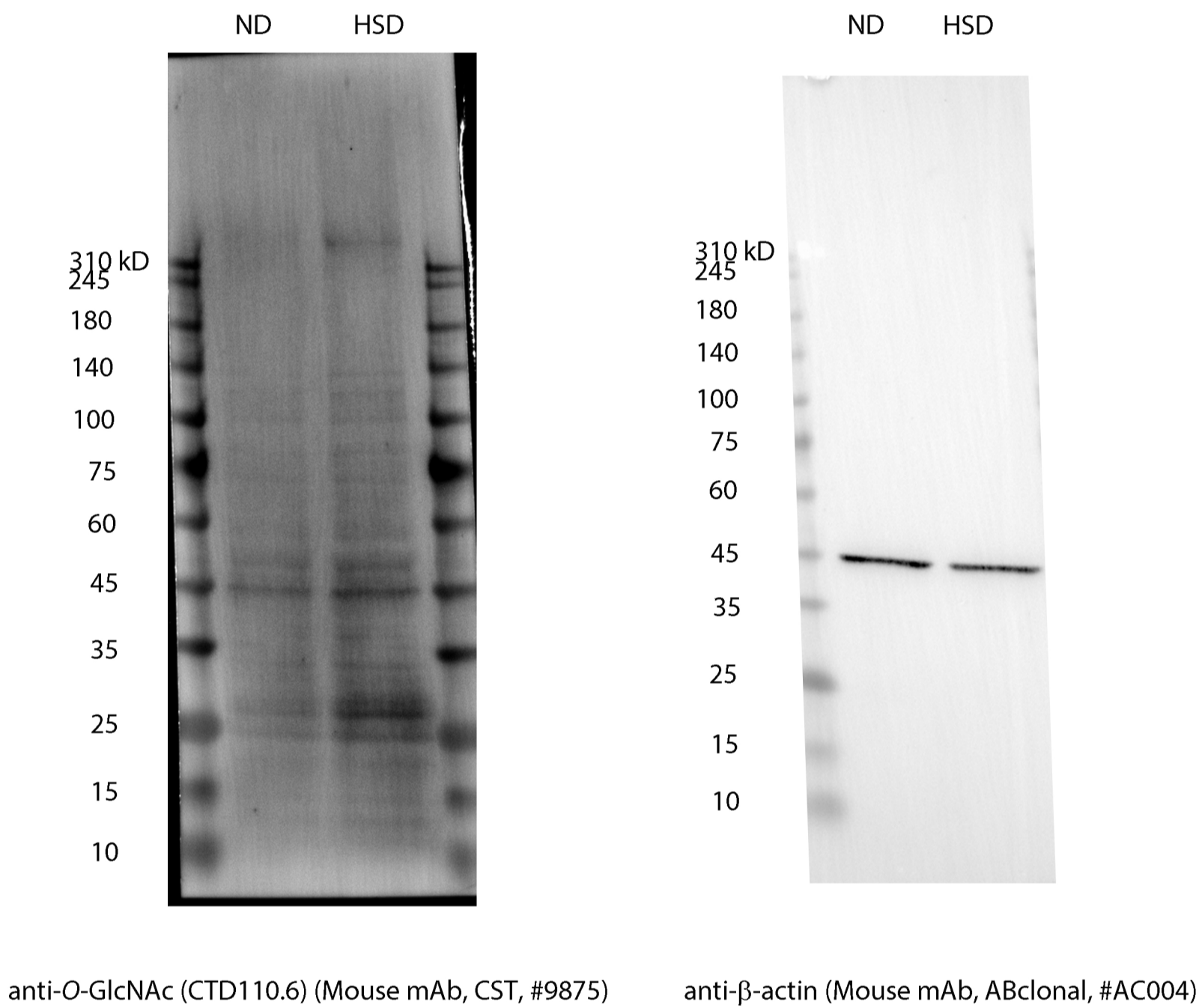

Supplementary Fig. 11b

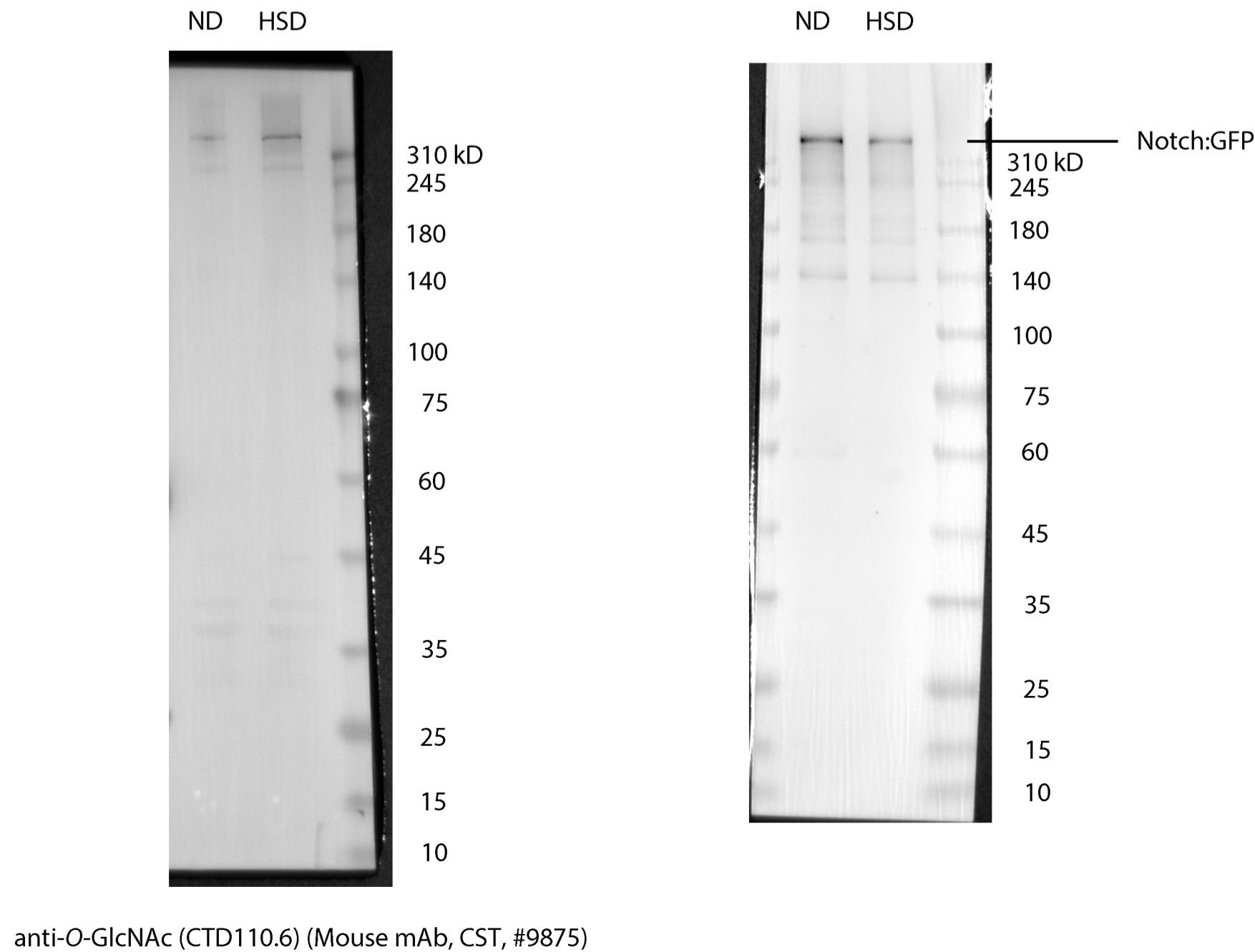

Supplement: Supplementary file 4 — Source data [file 41467_2024_46455_MOESM4_ESM.zip › Source Data/WB.pdf]
